# Lipid phase separation impairs membrane thickness sensing by the *Bacillus subtilis* sensor kinase DesK

**DOI:** 10.1128/spectrum.03925-23

**Published:** 2024-05-08

**Authors:** Margareth Sidarta, Ana I. Lorente Martín, Anuntxi Monsalve, Gabriela Marinho Righetto, Ann-Britt Schäfer, Michaela Wenzel

**Affiliations:** 1 Division of Chemical Biology, Department of Life Sciences, Chalmers University of Technology, Gothenburg, Sweden; 2 Centre for Antibiotic Resistance Research in Gothenburg (CARe), Gothenburg, Sweden; University of Manitoba, Winnipeg, Canada

**Keywords:** membrane fluidity, membrane thickness, DesK, daptomycin, antibiotics, membrane phase separation

## Abstract

Membrane fluidity and thickness have emerged as crucial factors for the activity of and resistance to several antimicrobials. However, the lack of tools to study membrane fluidity and, in particular, thickness in living bacteria limits our understanding of this interplay. The *Bacillus subtilis* histidine kinase/phosphatase DesK is a molecular sensor that directly detects membrane thickness. It controls activity of DesR, which regulates expression of the lipid desaturase Des, known for its role in cold adaptation and daptomycin susceptibility. We hypothesized that this property could be exploited to develop biosensors and reporters for antibiotic-induced changes in membrane fluidity and thickness. To test this, we designed three assays based on the *des* system: activation of the *Pdes* promoter as reporter for membrane thickening, localization of DesK-GFP(green-fluorescent protein) as proxy for rigidified membrane domains, and antibiotic sensitivity of *des*, *desK*, and *desR* deletion mutants as readout for the importance of membrane rigidification/thickening under the tested condition. While we could not confirm the suitability of the *des* system as reporter for antibiotic-induced changes in membrane thickness, we did observe that *des* expression is only activated by mild temperature shocks, likely due to partitioning of the sensor DesK into fluid membrane domains upon phase separation, precluding effective thickness sensing under harsh cold shock and antibiotic stress conditions. Similarly, we did not observe any sensitivity of the deletion mutants to either temperature or antibiotic stress, raising the question to what extent the *des* system contributes to fluidity adaptation under these conditions.

**IMPORTANCE:**

The *B. subtilis* des system is a prime model for direct molecular membrane thickness sensor and, as such, has been well studied *in vitro*. Our study shows that our understanding of its function *in vivo* and its importance under temperature and antibiotic stress is still very limited. Specifically, our results suggest that (i) the *des* system senses very subtle membrane fluidity changes that escape detection by established fluidity reporters like laurdan; (ii) membrane thickness sensing by DesK is impaired by phase separation due to partitioning of the protein into the fluid phase; and (iii) fluidity adaptations by Des are too subtle to elicit growth defects under rigidifying conditions, raising the question of how much the *des* system contributes to adaptation of overall membrane fluidity.

## INTRODUCTION

*Bacillus subtilis* is an important Gram-positive model organism. As ubiquitous soil bacterium, it is constantly exposed to changing environmental stress conditions like fluctuating nutrient supply, osmotic shifts, and temperature changes. To survive these challenges, *B. subtilis* possesses a number of adaptive stress response systems (1–8). One such mechanism is the homeoviscous adaptation of membrane fluidity during cold shock (4–6, 8).

Membrane fluidity describes the freedom of movement of proteins, lipids, and other constituents within the cell membrane (9). It is defined by the membrane components and thus depends on lipid head group composition, fatty acyl chain length, saturation, and branching, as well as membrane protein content, and correlates with membrane thickness (10–12). Membrane fluidity is also influenced by external factors, most prominently, temperature. A decrease in temperature leads to rigidification and concomitant thickening of the cell membrane, culminating in a phase change from liquid-crystalline to gel phase (5, 6).

Maintenance of membrane fluidity is critical for membrane function as a too rigid cell membrane limits the movement of constituents within the bilayer and disturbs cellular functions such as cell division, cell envelope synthesis, nucleoid replication and segregation, and membrane potential (13, 14). *B. subtilis* possesses two major mechanisms by which it adapts its membrane fluidity in response to temperature shifts: (i) the slower long-term adaptation of the overall content of branched-chain fatty acids and the specific ratio of *iso* and *anteiso* branched-chain fatty acids by *de novo* synthesis and (ii) the more rapid adaptation of the ratio of saturated and unsaturated fatty acids by desaturation of the fatty acyl chains of existing membrane lipids (6, 8).

**Fig 1:**
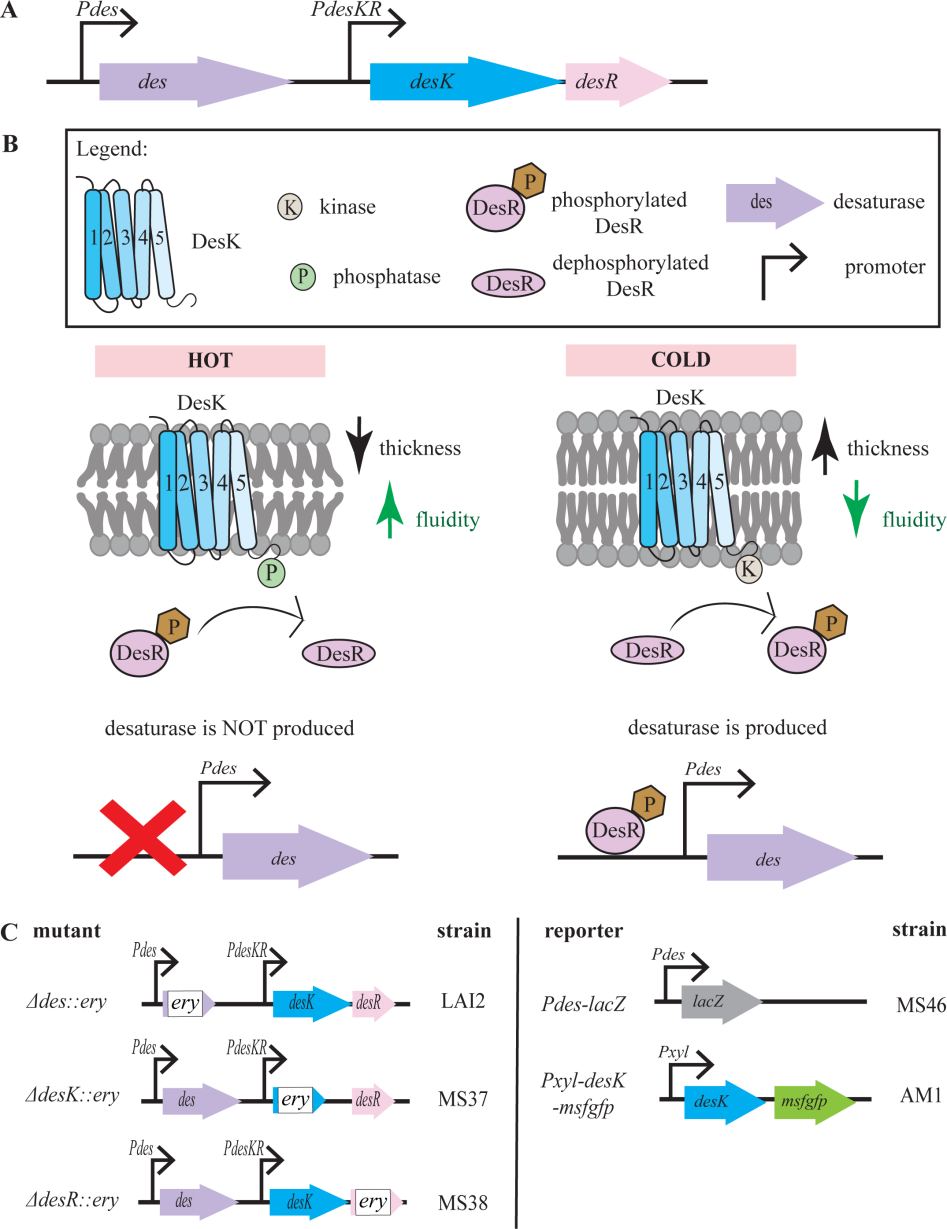
The *B. subtilis des* system. (A) Genetic organization of the *desKR* operon. (B) Current model of cold sensing by DesK. Figure adapted from Inda et al. (23). (C) Reporter strains used in this work. Gene deletions were based on strains published by Koo et al. (24), constructed by replacement of the deleted gene with an erythromycin resistance cassette keeping only the start and stop codons of the original gene. *msfgfp*, monomeric superfolder green-fluorescent protein.

The latter mechanism is mediated by the acyl lipid Δ5 desaturase Des, which is controlled by the two-component system DesKR (15, 16). The *desKR* operon is located directly downstream of the desaturase gene *des* (Fig. 1A). DesK is a dimeric, membrane-associated histidine kinase that possesses dual functionality as kinase and phosphatase, phosphorylating or dephosphorylating DesR in a temperature-dependent manner (15, 17, 18). DesK consists of a cytoplasmic kinase/phosphatase domain and five transmembrane domains, which were reported to be directly responsible for sensing changes in the physical properties of the cell membrane (13, 17–21). DesR is a DNA-binding response regulator (transcriptional activator) that regulates desaturase expression (15, 22).

The sensing mechanism of the *des* system has been characterized in a number of studies (5, 6, 8, 13, 15–19, 22, 25–27). The current model suggests that DesK detects changes in membrane fluidity through sensing bilayer thickness (Fig. 1B). Upon temperature decrease, the membrane rigidifies and increases in thickness, resulting in activation of the kinase-dominant state of DesK (21, 25). In this state, DesK forms a dimer, undergoes autophosphorylation of the conserved His188 in its catalytic domain, and consequently phosphorylates DesR (P-DesR) (18, 27). P-DesR forms a tetramer that binds to the *des* promoter (*Pdes*), activating expression of the *des* gene (22). Des then desaturates the fatty acyl chains of membrane lipids, resulting in membrane fluidization and concomitant decrease of bilayer thickness, which negatively regulates *des* expression by triggering the phosphatase activity of DesK. Consequently, DesK dephosphorylates P-DesR, and *des* transcription is stopped. This negative feedback loop ensures that lipid desaturation is shut off when sufficient fluidization has been achieved (15–17).

Indeed, *in vitro* studies on purified DesK reconstituted in liposomes have demonstrated that DesK detects membrane thickness changes caused by temperature variations (20, 23). Thus, the autokinase activity of DesK depends on fatty acyl chain length, with higher activity observed in membranes containing longer fatty acyl chains (20, 21). By quantifying the amount of ADP produced in the autophosphorylation reaction, Inda et al. could measure the kinase activity of reconstituted DesK and show that it senses temperature-dependent changes in membrane thickness (23).

Des not only has been shown to be induced upon cold shock (28) but also has been identified as a factor affecting the activity of the antibiotic daptomycin (29). This lipopeptide antibiotic has been shown to rigidify the cell membrane as part of its mechanism of action (30), matching well with the described function of Des. Membrane fluidity has also been shown to be a crucial factor for the mechanisms of action of several other membrane-targeting antibiotics (30–34), and some antimicrobial peptides have been shown to change membrane thickness *in vitro* (35–37). Yet, the importance of membrane fluidity and thickness for antibiotic action has not been thoroughly explored.

While membrane fluidity can be measured in living bacteria with different tools, the intimate link between fluidity and thickness makes it difficult to distinguish between the two. Since the proposed molecular mechanism of the sensor kinase DesK directly measures bilayer thickness, we reasoned that the *des* system could be used as a specific membrane thickness reporter. To assess this potential, we chose three assays based on the *des* system: activation of *Pdes* as reporter for membrane rigidification/thickening, localization of DesK as proxy for rigidified/thickened membrane domains, and antibiotic sensitivity of deletion mutants of *des*, *desK*, and *desR* (24) (see Fig. 1C for genetic constructs) to indicate whether rigidification/thickening plays a role in the compound’s activity. These assays were then tested under different temperature shift and antibiotic stress conditions.

Surprisingly, we could only activate the *des* promoter with a 2-h temperature shift from 37°C to 25°C, conditions under which we could not detect a significant change in membrane fluidity. Shorter or harsher shifts that resulted in measurable fluidity changes did not activate *Pdes*. When we examined the localization of DesK after temperature shifts and antibiotic treatment, we observed displacement of the GFP fusion protein into the fluid phase, which is counterintuitive, given the current model of the DesK-sensing mechanism. Finally, we did not observe any effects of deleting *des*, *desR*, or *desK* on the activity of antibiotics that affect membrane fluidity. Taken together, these findings indicate that the *des* system does not play a major role in antibiotic stress adaptation in *B. subtilis* and is not a suitable *in vivo* reporter for antibiotic-induced changes in membrane thickness. Our results raise important questions about the function of Des and its role in membrane fluidity adaptation.

## RESULTS

### Membrane fluidity

**Fig 2:**
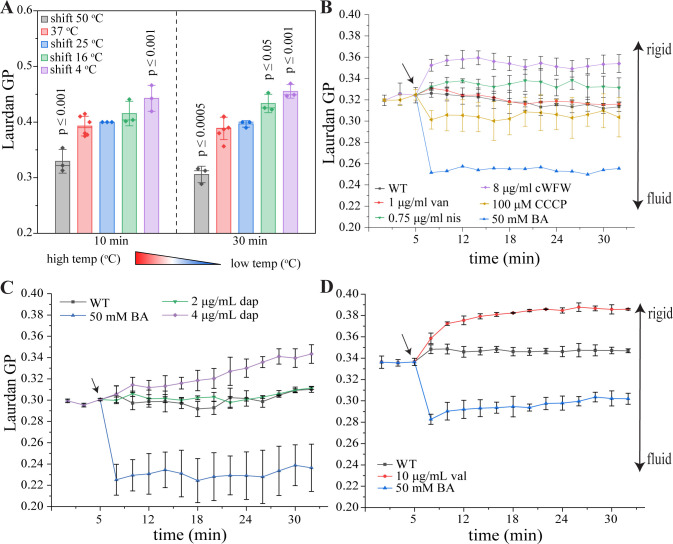
Membrane fluidity measurements using laurdan GP. An increase in GP indicates membrane rigidification, a decrease in fluidization. *B. subtilis* 168CA was grown at 37°C until early log phase (OD_600_ = 0.3) prior to (A) temperature shift or (B–D) antibiotic addition (nis, van, CCCP, cWFW, BA, dap, and val). Daptomycin (C) and valinomycin (D) require supplementation with 1.25-mM CaCl_2_ and 300-mM KCl, respectively, and thus have separate untreated and BA controls. Arrows indicate timepoints of antibiotic addition. Statistical significance in panel A was determined using two-tailed, homoskedastic *t*-tests. Only significant *P* values (≤0.05) are shown in the figure. Significance was tested between the 37°C sample and the shifted samples. BA, benzyl alcohol; CCCP, carbonyl cyanide m-chlorophenyl hydrazone; cWFW, cRRRWFW; dap, daptomycin; GP, generalized polarization; nis, nisin; val, valinomycin; van, vancomycin; WT, wild type.

Previous studies have used temperature shifts from 37°C to 13-18°C to activate the *des* system (28, 38–40). However, how these conditions relate to membrane rigidification and how they compare to antibiotic-induced changes in membrane fluidity are not clear. Therefore, we first measured changes in membrane fluidity after temperature shifts from 37°C to 25°C, 16°C, and 4°C, as well as after treatment with the rigidifying antibiotics daptomycin, valinomycin, nisin, and cWFW (a cyclic hexapeptide with the sequence cRRRWFW) (31, 41, 42). As additional controls, we included fluidizing conditions, namely, a temperature shift to 50°C and the fluidizing compounds benzyl alcohol and carbonyl cyanide m-chlorophenyl hydrazone (CCCP, a proton ionophore), as well as the cell wall synthesis inhibitor vancomycin, which does not affect membrane fluidity. Membrane fluidity was measured with laurdan, a fluorescent membrane dye that shifts its emission peak, depending on the amount of water molecules surrounding the probe (43). This shift allows calculation of generalized polarization (GP) values, which are indicative of membrane fluidity in terms of head group and fatty acyl chain spreading (44). As shown in Fig. 2, the selected conditions resulted in clear membrane fluidity changes after 10–30 min, with exception of the mildest temperature shift to 25°C, which did not significantly change membrane fluidity to a degree detectable with laurdan (Fig. 2A; Fig. S1). The selected antibiotic stress conditions resulted in different degrees and kinetics of membrane rigidification and fluidization (Fig. 2B through D), allowing thorough probing of the capacity of the *des* system as reporter for antibiotic effects on membrane thickness.

### Promoter activation

**Fig 3:**
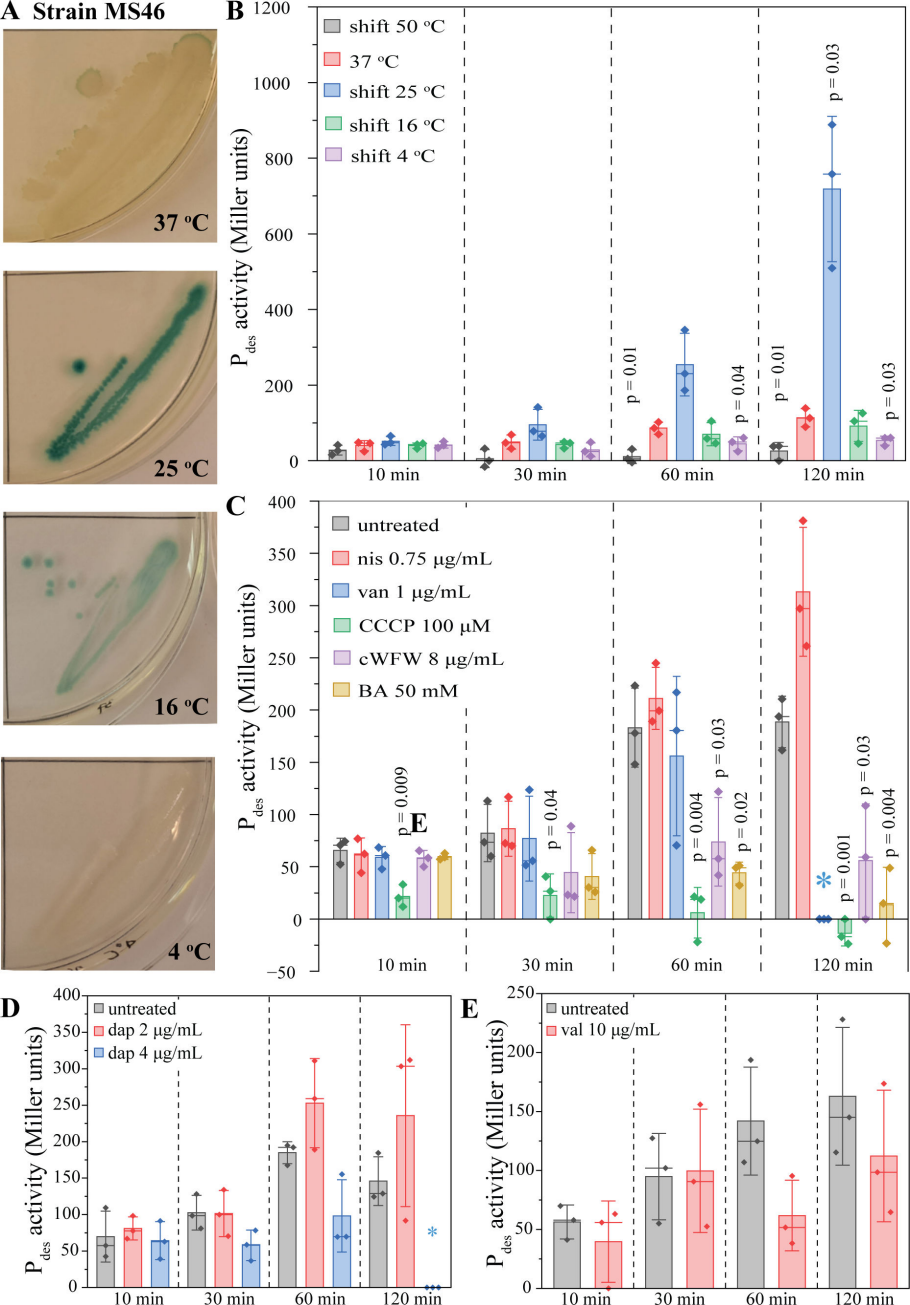
Promoter activation of *Pdes* measured by β-galactosidase activity using X-Gal (A) and *o*-nitrophenol-β-D-galactoside assays (B–E). (A) X-Gal assay. *B. subtilis* strains were grown at 37°C overnight and subsequently shifted to 25°C, 16°C, and 4°C, respectively. Pictures were taken 3 days after temperature shift (see Figure S2 for the full experiment). (B) Temperature shift experiments. *B. subtilis* MS46 (Pdes-lacZ) was cultured at 37°C and shifted to different temperatures during early log phase (OD_600_ = 0.3). (C–E) Antibiotic stress experiments. *B. subtilis* MS46 was grown until early log phase (OD_600_ = 0.3, 37°C) prior to addition of antibiotics (nis, van, CCCP, cWFW, BA, dap, and val). Daptomycin (D) and valinomycin (E) require supplementation with 1.25-mM CaCl_2_ and 300-mM KCl, respectively, and thus have separate untreated controls. Statistical significance was determined using two-tailed, heteroskedastic *t*-tests. Only *P* values of ≤0.05 are indicated. Blue asterisks indicate conditions for which no data could be obtained due to cell lysis. Significance was tested between the 37°C sample and the shifted samples and the antibiotic and corresponding untreated samples, respectively. BA, benzyl alcohol; CCCP, carbonyl cyanide m-chlorophenyl hydrazone; cWFW, cRRRWFW; dap, daptomycin; nis, nisin; val, valinomycin; van, vancomycin.

To assess induction of *des* expression, we used a strain carrying a promoter fusion of *Pdes* to the beta-galactosidase gene *lacZ* [strain MS46 *trpC2 amyE*::*Pdes*(–269 to +31)^a^-*lacZ*, in short *Pdes-lacZ*; see Fig. 1C] (15). We first checked induction of *Pdes* on agar plates containing X-Gal and indeed observed blue colonies after 3 days of shifting cultures grown at 37°C to 25°C and 16°C, whereby stronger induction was observed under the milder condition. Cultures shifted to 4°C underwent cell lysis and did not show a clear blue color (Fig. 3A; Fig. S2). This was in line with a previous study using the same LacZ reporter, where activation was observed after 36-h incubation at 25°C (15).

This slow response was surprising as the *des* system is considered a fast-responding adaptation mechanism (6, 8, 45), and at least some studies have shown rapid (≤ 1 h) induction of *des* expression after cold shock (4, 28). Therefore, we examined shorter temperature shock conditions using faster and more sensitive *o*-nitrophenol-β-D-galactoside (ONPG) assays (46, 47). Indeed, we could measure a significant (*P* = 0.03) increase of beta-galactosidase activity after 120 min after shifting the cultures to 25°C (Fig. 3B). Yet, we did not observe any significant promoter activation at earlier timepoints or with harsher cold shock conditions (16°C and 4°C), which was surprising, considering that (i) a shift to 25°C did not significantly alter membrane fluidity, not even after 120 min (Fig. 2A; Fig. S1), and (ii) a shift to 16°C is a commonly used condition to induce cold shock in *B. subtilis* and has been shown to activate *des* expression and DesK autophosphorylation in other studies (4, 20, 23, 28).

Despite this disparity, we proceeded to test *Pdes* activation by our selected antibiotic panel. However, no antibiotic stress condition led to a significant (*P* ≤ 0.05) induction of the *Pdes* promoter, though 120 min of nisin treatment came close (*P* = 0.06) (Fig. 3C through E). Additionally, the similarly rigidifying antibiotic cWFW significantly decreased beta galactosidase activity as did the fluidizing compounds benzyl alcohol and CCCP (Fig. 3C through E). Thus, we could not confirm a correlation between *Pdes* induction and membrane fluidity.

### DesK localization after temperature shift

Since *Pdes* activation was not a reliable reporter for antibiotic-induced fluidity changes, we examined whether the sensor kinase/phosphatase DesK could be used to assess membrane phase separation in *B. subtilis*. Fluorescent membrane dyes typically have a preference for the fluid phase and while some dyes are more sensitive to fluidity and can thus serve as reporters to a certain degree, there is no reliable proxy with a preference for the more rigid phase (43). Membrane proteins usually display similar behavior (30, 32–34). Yet, the current model of the DesK-sensing mechanism suggests that this protein should not partition into the fluid phase but rather into the rigid phase, or possibly be unaffected by phase separation. To examine this, we constructed a strain expressing DesK-GFP under a xylose-inducible promoter (strain AM1 *Pxyl-desK-msfgfp*, see Fig. 1C).

**Fig 4:**
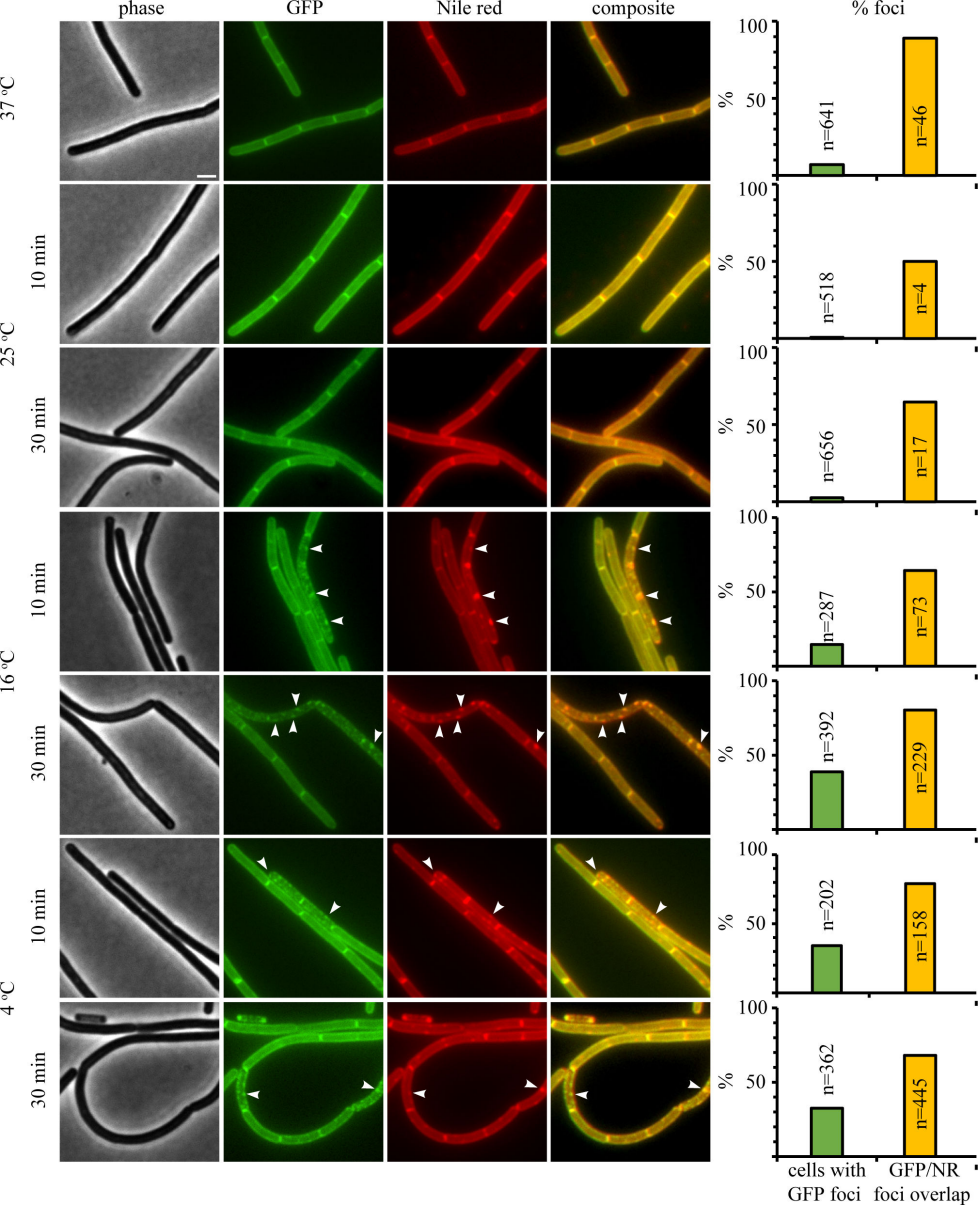
Localization of DesK after temperature shifts. *B. subtilis* AM1 (*Pxyl*-*desK*-*msfgfp*) was grown at 37°C until an OD_600_ of 0.3 and subsequently shifted to the indicated temperatures. Cells were stained with Nile red for 5 min prior to microscopy. Scale bar represents 2 μm. Arrowheads indicate GFP clusters overlapping with Nile red foci. Cells from three replicate experiments were pooled for quantification. *n* indicates the total number of counted cells or GFP foci, respectively.

As expected, DesK localized in the cell membrane and no change in localization was observed when cells were grown at different permissive temperatures (25°C, 30°C, 37°C, and 42°C) (Fig. S3). We then proceeded to characterize DesK localization after different temperature shifts. To this end, we stained cells with the fluorescent membrane dye Nile red, which stains cell membranes uniformly under normal culture conditions but partitions into fluid domains upon phase separation (43). As shown in Fig. 4, all temperature downshifts except 25°C induced Nile red to form clusters indicative of the formation of fluid membrane domains due to phase separation. Similarly, DesK-GFP clustered in membrane foci that overlapped with these domains, suggesting that it partitions into the fluid phase. Interestingly, we observed more GFP foci in response to heat than cold shock (Fig. S4). However, a much higher proportion of GFP foci co-localized with fluid Nile red foci in the cold shock (40%–50% for 16°C and 70%–80% for 4°C) than heat shock conditions (20%) (Fig. 4; Fig. S4), suggesting that, in the latter case, DesK may cluster independently of the formation of fluid membrane domains, e.g., due to protein aggregation.

To confirm these results, we stained AM1 with DilC12, a dye that has a high affinity for fluid membrane domains and is commonly used to visualize naturally occurring fluid membrane microdomains called regions of increased fluidity (RIFs) (48). DiIC12 detects membrane fluidity based on membrane thickness, a property that is mediated by its short hydrocarbon tail (43, 44, 49). Indeed, the majority of GFP foci co-localized with the DiIC12 dye (Fig. S5 and S6), confirming that DesK partitions into the fluid phase.

### DesK localization under antibiotic stress

**Fig 5:**
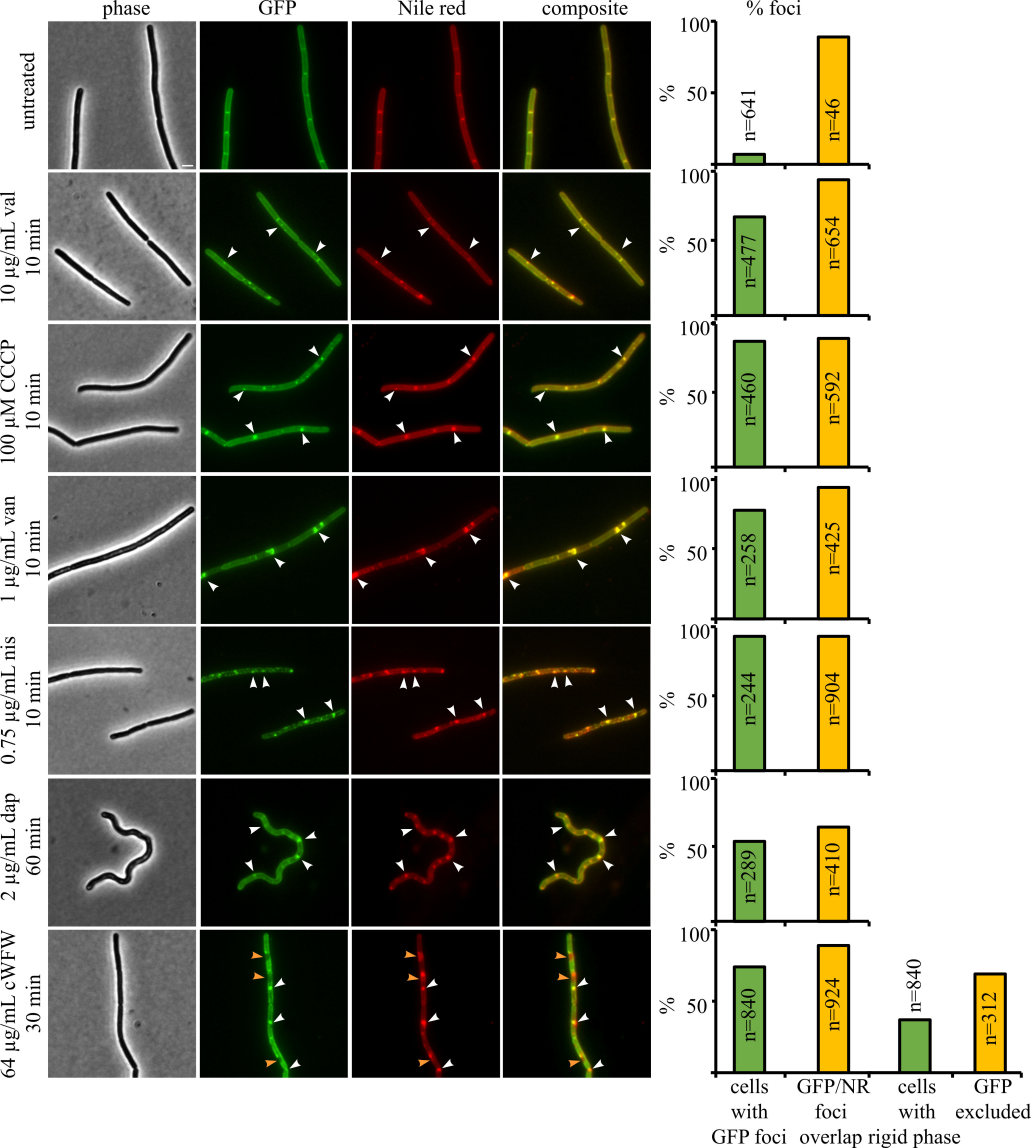
Localization of DesK under antibiotic stress. *B. subtilis* AM1 (*Pxyl*-*desK*-*msfgfp*) was grown at 37°C until an OD_600_ of 0.3 and subsequently treated with antibiotics (nis, van, CCCP, cWFW, dap, and val). Cells were stained with Nile red for 5 min prior to microscopy. Scale bar represents 2 μm. White arrowheads indicate GFP clusters overlapping with Nile red foci. Orange arrowheads mark rigid domains that are void of DesK-GFP but stained by Nile red. Cells from three replicate experiments were pooled for quantification. *n* indicates the total number of counted cells or GFP foci, respectively. CCCP, carbonyl cyanide m-chlorophenyl hydrazone; cWFW, cRRRWFW; dap, daptomycin; nis, nisin; val, valinomycin; van, vancomycin.

We then examined the localization of DesK under antibiotic stress conditions (Fig. 5). While daptomycin, nisin, cWFW, and valinomycin all have rigidifying effects on membrane fluidity when measured in batch (Fig. 1B and C), they have distinct effects on membrane phase separation when examined at single-cell level, as do the fluidizing compounds BA and CCCP.

Valinomycin and CCCP are ionophores that depolarize the cell membrane (50–52). Loss of membrane potential leads to delocalization of several peripheral membrane proteins (52). One of these membrane potential-dependent proteins is the actin homolog MreB, which organizes RIFs. Upon depolarization, MreB clusters at the cell membrane, concomitantly aggregating RIFs into large fluid domains that attract a number of membrane proteins (48). After treatment with both valinomycin and CCCP, DesK localized in distinct foci that almost perfectly overlapped with Nile red domains (Fig. 5; see Fig. S7 for additional controls and timepoints), suggesting that it behaves like most membrane proteins and partitions into the fluid phase.

Vancomycin, nisin, and daptomycin have in common the ability to cluster the cell wall peptidoglycan precursor lipid II (30, 53), which increases membrane fluidity and is associated with RIFs (30, 54, 55). Thereby, vancomycin’s effect on the cell membrane is limited to the formation of fluid lipid II clusters, while nisin uses lipid II as a docking molecule to form a transmembrane pore, additionally leading to intracellular content leakage (56). Daptomycin is thought to cluster lipid II similarly to vancomycin and nisin, yet these clusters are also enriched in phosphatidyl glycerol lipids and behave differently in that they do not generally attract membrane proteins (30, 57). Despite these differences, all three antibiotics caused clustering of DesK into foci that overlapped with Nile red domains (Fig. 5; see Fig. S7 for additional controls and timepoints).

The synthetic antimicrobial peptide cWFW is a special case that triggers large-scale membrane phase separation (31). The peptide shows two distinct phenotypes: (i) small fluid domains reminiscent of those caused by the clustering of RIFs and/or lipid II and (ii) large rigid domains that exclude certain membrane proteins while attracting others. Surprisingly, both types of domains can be visualized with Nile red (31). Here, we did indeed observe both phenotypes (Fig. 5; see Fig. S7 for timepoint). In cells showing fluid membrane clusters, DesK formed foci co-localizing with these domains. Conversely, in cells with large rigid domains, DesK was excluded from these areas. Both observations support our notion that DesK prefers fluid membrane regions.

We also examined cells treated with the membrane fluidizer benzyl alcohol yet did not observe a pronounced difference between the untreated and treated cells (Fig. S7), which was expected based on previous results (30).

### Antibiotic susceptibility

**Fig 6:**
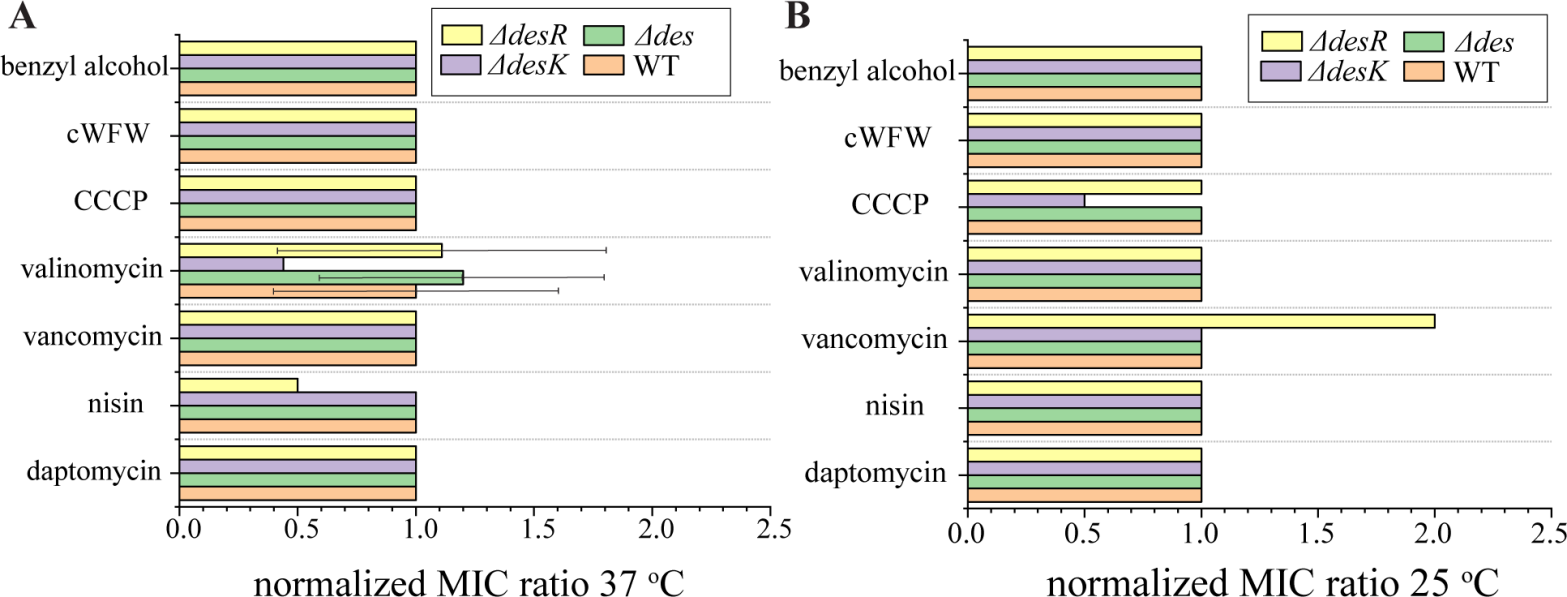
Minimal inhibitory concentrations of different antibiotics against *B. subtilis* 168CA (wild type) and the deletion mutant strains LAI2 (∆*des::ery*), MS37 (∆*desK::ery*), and MS38 (∆*desR::ery*) at 37°C (A) and 25°C (B). Values are expressed as fold change of the MIC value against the respective mutant compared with the wild type. Absolute values are listed in Table S1. Error bars represent relative standard deviations. Where no error bar is shown, replicate values were identical.

A previous study showed that a strain lacking the desaturase Des was slightly more susceptible to daptomycin at 24°C (29). Therefore, we determined the minimal inhibitory concentrations (MICs) of the different antibiotics against the wild type (168CA) and the single-deletion mutants of *des* (LAI2), *desK* (MS37), and *desR* (MS38), both at 37°C and 25°C (Fig. 6; Table S1). None of the tested antibiotics showed clearly different MICs when comparing the mutants with the wild type. The only differences observed (valinomycin and nisin at 37°C and CCCP and vancomycin at 25°C) were within a twofold MIC range, which is within the normal error of microdilution assays using serial twofold dilutions. Therefore, we performed acute shock experiments, which often display more subtle differences in activity than MIC assays. To this end, mid-log phase cells were challenged with different antibiotic concentrations and growth curves were recorded by optical density (OD) measurements (Fig. S8 to S21). Only with daptomycin did we observe slight differences in growth and only at 4 µg/mL, which normally leads to extended initial cell lysis before cultures resume normal growth (Fig. S12). This initial cell lysis was absent in the *des* and *desR* deletion strains, yet this effect was only observed at 37°C, and no pronounced growth differences were observed for any of the remaining conditions (Fig. S8 to S12, 14–21).

**Fig 7:**
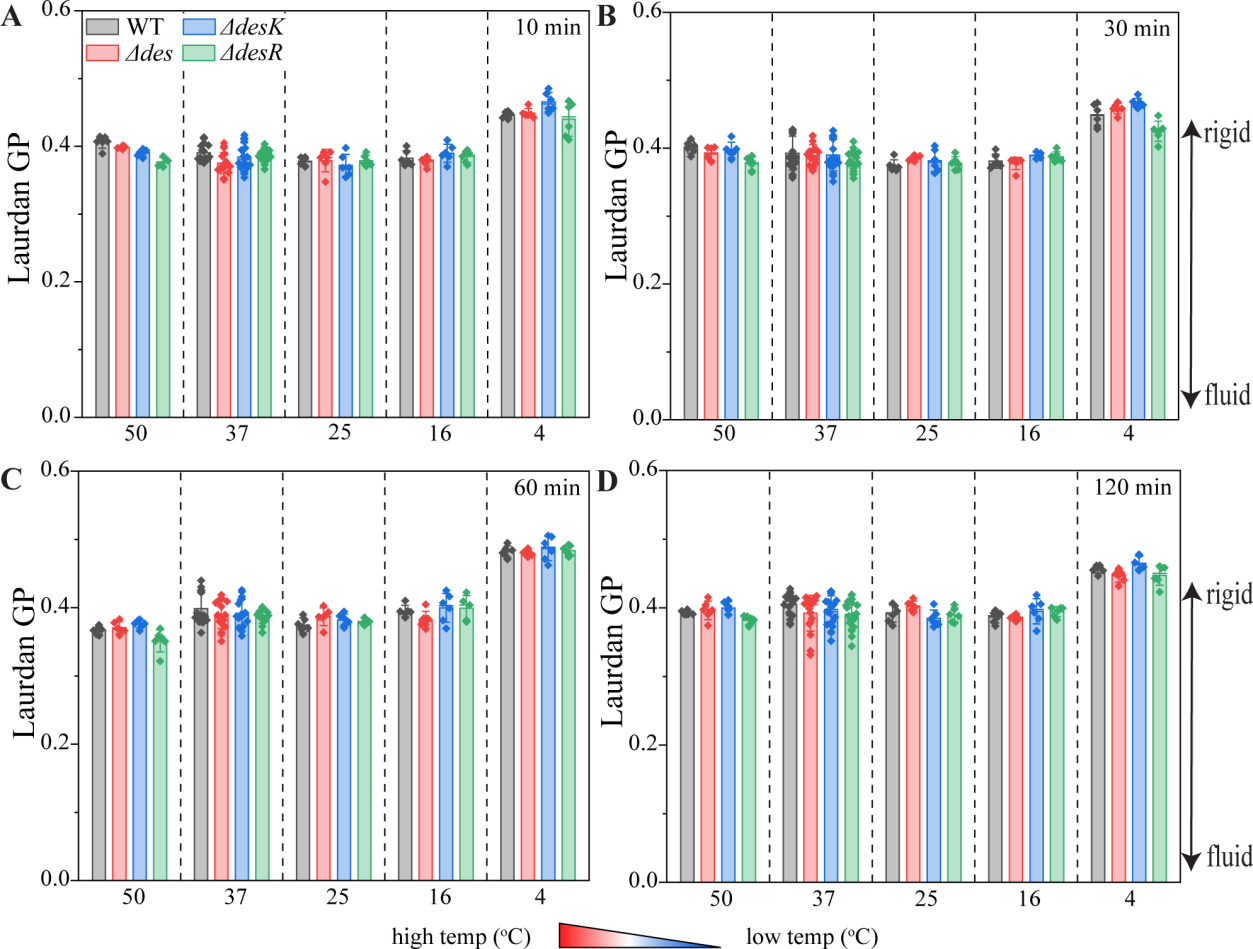
Membrane fluidity of deletion mutants after temperature shifts for 10 (A), 30 (B), 60 (C), and 120 min (D). *B. subtilis* 168CA (wild type), LAI2 (∆*des::ery*), MS37 (∆*desK::ery*), and MS38 (∆*desR::ery*) were grown at 37°C until log phase prior to laurdan staining and temperature shifting. Error bars represent standard deviations of the mean. Statistical significance was determined using two-tailed, homoskedastic *t*-tests but no significance (*P* ≤ 0.05) was observed.

Taken together, it seems as if the *des* system does not play a role in adaptation to antibiotic stress. Since we only observed promoter activation under conditions that did neither alter overall membrane fluidity (Fig. 2) nor induce phase separation (Fig. 4), we wanted to know if the *des* system notably contributes to membrane fluidity at all. To this end, we measured the laurdan GP of the wild type and the deletion mutants. We did not observe any notable difference in membrane fluidity between the mutants and the wild type when constantly grown at 37°C and 25°C (Fig. S22). Likewise, the membrane fluidity of the mutants did not notably differ from the wild type after temperature shifts from 37°C to 4°C, 16°C, 25°C, and 50°C (Fig. 7), suggesting that the *des* system does not contribute to fluidity adaptation to a degree that can be measured with laurdan.

## DISCUSSION

### Suitability of the *des* system as reporter for antibiotic studies

Membrane fluidity and thickness have emerged as important factors for the mechanisms of action of several membrane-targeting antibiotics (30–34, 36, 37). While membrane fluidity in terms of lipid head group and fatty acyl chain spreading can be measured with fluorescent dyes like laurdan, tools for assessing membrane thickness are limited, and distinguishing these two factors in the living bacterium is near impossible. The proposed sensing mechanism of DesK is specifically based on sensing membrane thickness (20, 23); hence, we hypothesized that the *des* system could possibly be used as a tool to detect membrane thickness changes *in vivo*.

To test this, we employed three approaches, *Pdes* promoter activity, DesK-GFP localization, and susceptibility of deletion mutants of *des*, *desK*, and *desR*. However, none of the tested antibiotics significantly activated the *Pdes* promoter (Fig. 2B through D) and none of the deletion mutants displayed increased sensitivity toward any of the tested compounds (Fig. 6; Fig. S8 to S21). While antibiotic effects on DesK localization were observed, they were consistent with partitioning of DesK into the fluid phase upon clustering of fluid membrane domains (i.e., RIFs) or phase separation (Fig. 5; Fig. S7). This is the same phenotype most membrane proteins exhibit and not indicative of rigidified or thickened domains, as hypothesized. These results show that none of the three assays is suitable for reporting on membrane fluidity or thickness changes induced by antibiotic exposure.

It should be mentioned that some significant differences were observed in the different assays. Thus, CCCP (membrane depolarizing and fluidizing), benzyl alcohol (fluidizing), and cWFW (transiently depolarizing, rigidifying, and inducing large-scale phase separation) reduced *Pdes* promoter activity in ONPG assays (Fig. 2B). Yet, these results do not correlate with membrane fluidity changes induced by these antibiotics or any of their other known effects, precluding drawing conclusions from these results. Similarly, the *des* and *desR* deletion strains were slightly more resilient against daptomycin in acute shock experiments, yet only at 37°C and not 25°C (Fig. S12). This is counterintuitive since membrane rigidification has been proposed to be a significant part of daptomycin’s mode of action (30), and we expected to see increased sensitivity of the mutants. Indeed, Hachmann et al. have observed that a *des* deletion strain is slightly more sensitive to daptomycin, yet only at 24°C and not 37°C (29). However, here we could not reproduce these results, neither in MIC nor in acute shock assays. To exclude that there is a difference between the deletion strains, we compared the MICs of our ∆*des::ery* strain (LAI2) with the ∆*des::spc* strain used by Hachmann *et al*. (HB5134) at 24°C and 37°C (29). Yet, we did not observe a difference in daptomycin MIC with either mutant (Fig. S23). Since Hachmann *et al*. observed only a minor decrease in MIC (1.75-fold; for comparison, a strain depleted of phosphatidyl glycerol shows an 8-fold MIC increase) (29), it is conceivable that the effect of Des on daptomycin activity is minor, which would align with our results.

### Implications for *des* sensing and function under cold shock

As controls for our antibiotic assays, we conducted temperature shift experiments. Surprisingly, these resulted in several unexpected observations, raising questions about the role of the *des* system during cold shock, specifically about the extent of membrane rigidification needed for *Pdes* activation, induction time, and effects on membrane fluidity. Thus, we tested temperature shifts from 37°C to 25°C, 16°C, and 4°C. Both 37°C–25°C and 37°C–16°C are common temperature shift conditions used to study cold adaptation in *B. subtilis* (15, 20, 23, 28, 38–40, )*,* while 4°C leads to cell death by autolysis (58–60) and can therefore only be used for short shifting experiments. In our laurdan-based fluidity measurements, only the shifts to 16°C and 4°C resulted in significant membrane rigidification (Fig. 2A). However, only the shift to 25°C resulted in activation of *Pdes* (Fig. 3B). This effect was well reproducible and in line with previous experiments using the same *Pdes-lacZ* construct (15) as well as *in vitro* studies on purified DesK reconstituted in liposomes, which have demonstrated that DesK detects membrane thickness changes when shifted from 37°C to 25°C (20, 23).

However, it prompts the question why specifically the condition that did not induce measurable fluidity changes induced *Pdes* but not the harsher temperature shifts. Our results suggest that DesK only senses minute changes in membrane thickness and does not get activated upon stronger membrane rigidification. However, when performed on solid medium, *Pdes* was also activated by shifting to 16°C, even though β-galactosidase activity was lower than at 25°C and took 3 days to become visible (Fig. S2). This effect can possibly be explained by the observed phase separation effects. The proposed sensing mechanism of DesK would require the protein to not move into the fluid phase, but either stay evenly distributed or reside in rigidified membrane areas. However, we observed a clear preference of DesK-GFP to partition into the fluid phase under all tested conditions (Fig. 4 and 5; Fig. S4 to S7). Thus, we hypothesize that (i) *Pdes* is already activated by minor changes in membrane fluidity/thickness that escape detection by laurdan GP measurements and (ii) that this sensing is disturbed by phase separation due to partitioning of DesK into the fluid phase. It is further conceivable that phase separation effects are eventually counteracted by other means of fluidity adaptation, such as *de novo* synthesis of branched and short-chain fatty acids, possibly explaining the late and lesser activation of *Pdes* observed on plate after several days of incubation.

These hypotheses would also explain why *Pdes* was not activated by any of the tested rigidifying antibiotics, which all lead to some degree of phase separation (Fig. 5; Fig. S7). An alternative explanation could be that antibiotics that interfere with membrane integrity, such as valinomycin, CCCP, nisin, and cWFW, may also alter intracellular pH. Recently, it has been reported that DesK is able to sense pH and becomes inactive at low pH regardless of temperature (61), suggesting that activation of *des* system is not as simple as previously thought.

Another curious observation was the slow kinetics of *Pdes* activation. On X-Gal plates, it took 3 days to observe blue colonies (Fig. S2), which was consistent with a previous study, where an activation time of 36 h was observed with the same LacZ reporter (15). Even in the faster and more sensitive ONPG assays, it took 120 min to observe significant promoter activation (Fig. 3B). A *B. subtilis* transcriptomic profiling study used 60-min incubation at 18°C to induce cold shock (shifted from 37°C). Under these conditions, a 1.7-fold induction of *des* was observed (28). This is in contrast to our results that did not show activation in this timeframe. Yet, a 1.7-fold change in transcript levels may not be sufficient to result in significant changes in β-galactosidase activity. While these experiments are not fully comparable since transcriptomics were performed in minimal medium, it is possible that stronger induction may have been observed with a milder and longer cold shock as well.

Generally, the *des* system is described as a fast-reacting response system, modifying already existing membrane lipids in an “emergency fluidization response,” which is opposed to fluidization through *de novo* synthesis of fatty acids (6, 8, 62, 63). In previous antibiotic stress experiments, one doubling time [30 min in Luria-Bertani (LB), 30°C] was sufficient to detect major changes in the ratio of branched and unbranched fatty acids as well as the length of fatty acyl chains (33). This suggests that *de novo* synthesis is in fact acting faster than the *des* system.

Lastly, we could not detect any phenotype of the *des*, *desK*, and *desR* deletion mutants. While our hypothesis that phase separation interferes with DesK sensing would explain why no effects on antibiotic activity were observed (Table S1; Fig. 6), it was surprising that none of the mutants appeared to be defective in fluidity adaptation (Fig. 7). Assuming that the thickness changes detected by DesK are too subtle to be detected by laurdan, it could be conceivable that the effects of Des activity may likewise escape detection in this assay. However, it should be noted that laurdan has been shown capable of detecting fluidity changes caused by inhibition of desaturase activity in mammalian cells, indicating that it is in principle suitable to detect differences in desaturation (64). Thus, our observations raise the question how strong the impact of the *des* system on fluidity adaptation really is. Thus, *B. subtilis* possesses 80%–96% branched-chain fatty acids (33, 65, 66), and its main regulation mechanism for membrane fluidity is the branched-chain fatty acid content and the ratio of *iso* and *anteiso* branched-chain fatty acids (14, 67). *B. subtilis* grown in LB only possesses 5%–6% straight-chain fatty acids with a 0.075 ratio of unsaturated to saturated fatty acids (33). Thus, it appears that fatty acid desaturation plays a minor role in overall membrane fluidity adaptation. This is consistent with a lack of any growth defect of a *des* mutant at lower temperature (Figures S8 to S21) (14).

### Conclusion

In conclusion, we could not confirm that the *des* system can be used as reporter for antibiotic-induced fluidity changes and is limited even in reporting on temperature-induced changes. From our results, we hypothesize that (i) the *des* system senses membrane fluidity changes that are much more subtle than those induced by antibiotics and escapes detection by laurdan GP measurements; (ii) that membrane thickness sensing by DesK is impaired by phase separation due to partitioning of the sensor kinase/phosphatase into the fluid phase; and (iii) that the fluidity adaptations carried out by Des are too subtle to be detected by laurdan and to elicit growth defects under rigidifying conditions, raising the question of how important the contribution of the *des* system to fluidity adaptation is. Future studies will be needed to further address these questions.

## MATERIALS AND METHODS

### Strain construction

Strains, plasmids, and primers are listed in Tables S2 and S3. *Escherichia coli* strains used as cloning hosts were grown at 37°C in LB medium or on LB agar. *B. subtilis* strains were grown at 37°C in Spizizen minimal medium (SMM) (68), LB medium, or on LB agar. Where appropriate, selection antibiotics were added at the following concentrations: 100-µg/mL ampicillin, 2-µg/mL erythromycin, and 100-µg/mL spectinomycin.

### Plasmid construction

The *desK* gene sequence was obtained from the SubtiWiki database (69). Chromosomal DNA isolation was performed following standard phenol-chloroform extraction (70). Plasmid pAM1 (*Pxyl-desK-msfgfp*) was designed in SnapGene, version 6.2 (www.snapgene.com), and constructed with Gibson assembly (71) (Gibson Assembly Cloning Kit, NEB). The *desK* gene was amplified from *B. subtilis* 168CA chromosomal DNA using the primer pair MSP11/MSP93. The pMW1 plasmid backbone was linearized by PCR using primers MWP1/Abs1. After DpnI treatment (37°C for 1 h) and subsequent purification, Gibson assembly was performed using a 1:2 (0.02 pmol:0.04 pmol) vector-to-insert ratio, resulting in plasmid pAM1, which was then transformed into chemically competent *E. coli* (72). The plasmid was isolated and verified by sequencing (Eurofins MWG).

### Construction of *B. subtilis* strains

Strains were constructed by transforming either plasmid or chromosomal DNA into *B. subtilis* 168CA as indicated in Table S2. *B. subtilis* was grown to natural competence using a standard starvation protocol (73). Briefly, *B. subtilis* 168CA cells were grown overnight in competence medium [fully supplemented SMM containing 0.5% (wt/vol) glucose, 6-mM MgSO_4_, 2.4-mM tryptophan, 0.02% casein hydrolysate, and 0.0011% Fe-NH_4_-citrate]. Overnight cultures were diluted 1:10 in fresh competence medium and grown for 3 h prior to 1:1 dilution in starvation medium (SMM supplemented with only 0.5% glucose and 6-mM MgSO_4_). Cells were sufficiently competent after 1.5 h of starvation. Aliquots of competent cells were mixed with 1 µg/mL of DNA and incubated for 1 h prior to plating on selective LB agar plates. GFP and LacZ reporter strains were screened for double crossover insertion into the *amyE* locus by testing amylase activity. To this end, colonies were grown on LB agar supplemented with 0.5% (wt/vol) starch and covered with Lugol’s solution. Correct transformants were unable to produce amylase and were non-transparent after addition of Lugol’s solution. All strains were confirmed by PCR (see Fig. S23 for PCR confirmation of deletion mutants).

### Stress experiments

For all stress experiments, *B. subtilis* strains were grown in LB at 37°C under constant agitation. Overnight cultures were diluted 1:100 in fresh LB and grown to an OD_600_ of 0.3 before being subjected to the respective stress conditions. Shaking was maintained under all conditions to ensure sufficient aeration. For AM1, expressing DesK-msfGFP from the xylose-inducible *Pxyl* promoter, the medium was supplemented with 0.1% (wt/vol) xylose.

#### Temperature shock experiments

For temperature shock experiments, cultures were grown at 37°C until reaching an OD_600_ of 0.3 (or adjusted to an OD_600_ of 0.3 for laurdan and DiIC12 experiments) and subsequently shifted to 50°C, 25°C, 16°C, or 4°C using a temperature-controlled thermoblock. An untreated control was maintained at 37°C. Samples were taken after 10 and 30 min and, where necessary, 60 and 120 min.

#### Antibiotic treatment

For antibiotic treatment, cells were grown at 37°C until reaching an OD_600_ of 0.3 and were subsequently treated with subinhibitory concentrations of antibiotics as determined in previous studies and specified in Table 1 (30, 33, 52, 74–76) (see Fig. S26 for growth inhibition). For daptomycin treatment, the growth medium was supplemented with 1.25-mM CaCl_2_ (29). For valinomycin, a modified growth medium (LB containing 300-mM KCl instead of NaCl) was used (77). Untreated controls were examined for each growth medium.

**TABLE 1:** Experimental conditions of antibiotic treatment done in this work

Antibiotic	Target/mechanism	Concentration	Timepoint(s)
Daptomycin	Membrane, lipid II	2 µg/mL	60 min
Nisin	Membrane, lipid II	0.75 µg/mL	10 min
Vancomycin	Lipid II	1 µg/mL	10 and 30 min
Valinomycin	Potassium ionophore	10 µg/mL	10 min
CCCP	Proton ionophore	100 µM	10 and 30 min
cWFW	Membrane phase separation	64 µg/mL	10 and 30 min
Benzyl alcohol	Membrane fluidization	50 mM	10 min

### Laurdan spectroscopy

Laurdan generalized polarization was measured in a BMG Clariostar Plus plate reader as described previously (44, 78). For daptomycin and valinomycin samples as well as their respective untreated controls, laurdan buffer was supplemented with 1.25-mM CaCl_2_ or 300-mM KCl, respectively. Kinetic measurements after antibiotic addition were performed as described by Schäfer et al. (78). For temperature shock conditions, endpoint measurements were performed as described in the same reference with the following modifications: cells were grown at 37°C in LB medium supplemented with 0.2% glucose. After reaching an OD_600_ of 0.6, cultures were stained with 10-µM laurdan for 5 min, washed four times with prewarmed (37°C) laurdan buffer[(phosphate-buffered saline, 0.2% glucose, and 1% DMF(dimethylformamide)], and resuspended to an OD_600_ of 0.3 using the same buffer. Washed cells were then shifted to 50°C, 25°C, 16°C, or 4°C, or maintained at 37°C as control. Laurdan fluorescence was measured 10 and 30 min (and 60 and 120 min, where indicated) after shifting to the new temperature.

### Promoter induction assays

For promoter activation on agar plates, *B. subtilis* MS46 (*Pdes-lacZ*) was spread on LB agar supplemented with 100-µg/mL X-gal and incubated overnight at 37°C. Plates were then shifted to 25°C, 16°C, or 4°C and regularly checked for the appearance of blue color around the colonies. Pictures were taken after 3 days when the blue color was visible by eye. For ONPG assays, *B. subtilis* MS46 was exposed to temperature and antibiotic stress conditions as described above. β-Galactosidase activity was measured according to published procedures (46, 47) with minor modifications. Briefly, treated cells were centrifugated at 16,100 × *g* for 2 min. Cell pellets were resuspended in 100 µL of Z-buffer (0.1-M Na_2_HPO_4_, 0.5-M NaH_2_PO_4_, 10-mM KCl, 2-mM MgSO_4_, and 0.27% β-mercaptoethanol). Aliquots were transferred to a flat-bottom microplate, and the OD_600_ was recorded using a Clariostar Plus plate reader. Subsequently, 73.5 µL of Z-buffer and 0.5 µL of 20-mg/mL lysozyme solution were added to each well. The plate was then incubated at 37°C for 20 min, followed by addition of 1-µL Triton X-100 (10%), 75.2 µL of Z-buffer, and 24.8 µL of 60-mM ONPG to each well. The OD_420_ and OD_550_ were read every 30 s for at least 5 min. The angular coefficient (*α*) of the linear plot (equation 1) was determined using Origin, version 2023 10.0.0.154. Using equation 2, we obtained activation of the *des* promoter in Miller units. For each biological replicate, the associated error was obtained by propagation of the *α* standard error.


(1) $$OD_{420}-\left(1.75\times OD_{550}\right)=\alpha \times {\text{time}}_{\text{min }}+\beta$$

(2) $$M.U.\;=\;\frac{1000\;\times \;\alpha }{0.1\;\times \;OD_{600}}$$

### Fluorescence microscopy

For general membrane staining, cells were stained with 1-µg/mL Nile red for 5 min immediately prior to microscopy. DiIC12 staining was performed immediately prior to temperature shifting, following a previously published protocol (44, 49). Samples (0.5 µL) were spotted on glass slides covered with a thin film of 1.2% agarose, covered with a poly-L-dopamine-coated coverslip (79), and imaged using a Nikon Eclipse Ti2 inverted fluorescence microscope equipped with a CFI Plan Apochromat objective (DM Lambda 100× Oil N.A. 1.45, W.D. 0.13 mm, Ph3), a Lumencor Sola SE II FISH 365 light source, a Photometrics PRIME BSI camera, an Okolab incubator, and Nis ELEMENTS AR software, version 5.21.03. Images were processed and analyzed with Fiji (80). For counting phenotypes, cells were straightened and segmented using the ObjectJ plugin ChainTracer (81). Foci were assessed and counted manually as described previously (76). For co-localization, all foci in the GFP channel were visually identified and mapped. Then, channels were compared, and those GFP foci that overlapped with Nile red foci were counted as “overlapping” and expressed as percentage of all GFP foci. Lysed cells were excluded from data analysis. Cell lysis was defined as loss of phase density.

### MIC

MICs against *B. subtilis* 168CA, LAI2, MS37, MS38, HB5134, and TB92 were determined in a standard microdilution assay (33), following the guidelines issued by the Clinical Laboratory Standardization Institute (82), with the exception of using LB as culture medium. Samples were incubated at 24°C, 25°C, or 37°C for 16 h, and growth was monitored continuously by following the OD_600_ in a BMG Clariostar Plus plate reader. The lowest antibiotic concentration inhibiting visible growth was defined as MIC. The experiment was done in a minimum of two biological replicates.

### Acute shock experiments

Overnight cultures of *B. subtilis* 168CA, LAI2, MS37, and MS38 were diluted to an OD_600_ of 0.06–0.1 in fresh LB medium, followed by transfer of 190 µL of the diluted cultures to sterile 96-well plates. Cultures were incubated at 25°C or 37°C under constant shaking in a BMG Clariostar Plus plate reader. After reaching an OD_600_ of 0.3, 10 µL of the respective antibiotics was added to the cells (Table 1), and OD measurements were continued until the untreated control reached stationary growth phase.

### Statistical analysis

Unless stated otherwise, experiments were performed in at least biological triplicates, and numerical values represent the average of independent replicate experiments. Error bars represent standard deviations. For quantification of microscopy experiments, images of three biological replicates were pooled for each sample. To test statistical significance, *P* values were calculated with either heteroscedastic or homoscedastic *t*-tests as indicated in the figure legends using OriginPro (OriginLab Corporation, versions 2022b and 2023).

## SUPPLEMENTAL MATERIAL

Supplemental material spectrum.03925-23-s0001.pdf Tables S1-S3; Fig. S1-S26.

ASM does not own the copyrights to Supplemental Material that may be linked to, or accessed through, an article. The authors have granted ASM a non-exclusive, world-wide license to publish the Supplemental Material files. Please contact the corresponding author directly for reuse.
