# Supplementary material for: Lipid phase separation impairs membrane thickness sensing by the Bacillus subtilis sensor kinase DesK
Source: Microbiol Spectr. 2024 May 8;12(6):e03925-23. doi: 10.1128/spectrum.03925-23 (PMC11237406; doi:10.1128/spectrum.03925-23)
Supplement: Supplemental material — Tables S1-S3; Fig. S1-S26. [file spectrum.03925-23-s0001.pdf]

Supplementary Information

Lipid phase separation impairs membrane thickness sensing by the *Bacillus subtilis* sensor kinase DesK

Margareth Sidarta[1,2], Ana I. Lorente Martín[1#§], Anuntxi Monsalve[1¤§], Gabriela Marinho Righetto[1,2], Ann-Britt Schäfer[1,2], Michaela Wenzel[1,2]*

[1]Division of Chemical Biology, Department of Life Sciences, Chalmers University of Technology, 412 96 Gothenburg, Sweden

[2]Centre for Antibiotic Resistance Research in Gothenburg (CARe), Gothenburg, Sweden

[#]Current address: Department of Development and Regeneration, University of Leuven (KU Leuven), 3000 Leuven, Belgium

[¤]Current address: Sahlgrenska Center for Cancer Research, Department of Microbiology and Immunology, University of Gothenburg, 413 90 Gothenburg, Sweden

[§]These authors contributed equally to this work.

*Corresponding author: Michaela Wenzel, Division of Chemical Biology, Department of Life Sciences, Chalmers University of Technology, Kemigården 4, 412 96 Gothenburg, Sweden; Phone: 0046 31 772 2074; Email: wenzelm@chalmers.se

Supplementary tables

Table S1: Minimal inhibitory concentrations of different antibiotics against *B. subtilis* wild type (WT) and deletion mutants at 37 and 25 ºC. 4
Table S2: Strains and plasmids used in this work. 5
Table S3: Primers used in this work. 6

Supplementary figures

Figure S1: Membrane fluidity measurements using laurdan generalized polarization (GP) spectroscopy 7
Figure S2: *Pdes* promotor activation measured by β-galactosidase activity on agar plates supplemented with X-gal 8
Figure S3: Localization of DesK-GFP at different permissive temperatures 9
Figure S4: Localization of DesK after temperature shift to 50 °C 10
Figure S5: Co-localization of DesK with DiIC12 after temperature shock 11
Figure S6: Co-localization of DesK with DiIC12 after temperature shock to 50 °C 12
Figure S7: Localization of DesK after antibiotic treatment (additional controls and timepoints) 13
Figure S8: Growth curves of 168CA (wild type), LAI2 (*Δdes*), MS37 (*ΔdesK*), MS38 (*ΔdesR*) after acute shock with different concentrations of valinomycin (val) at 37 ºC 14
Figure S9: Growth curves of 168CA (wild type), LAI2 (*Δdes*), MS37 (*ΔdesK*), MS38 (*ΔdesR*) after acute shock with different concentrations of carbonyl cyanide m-chlorophenylhydrazone (CCCP) at 37 ºC 15
Figure S10: Growth curves of 168CA (wild type), LAI2 (*Δdes*), MS37 (*ΔdesK*), MS38 (*ΔdesR*) after acute shock with different concentrations of vancomycin (van) at 37 ºC 16
Figure S11: Growth curves of 168CA (wild type), LAI2 (*Δdes*), MS37 (*ΔdesK*), MS38 (*ΔdesR*) after acute shock with different concentrations of nisin (nis) at 37 ºC 17
Figure S12: Growth curves of 168CA (wild type), LAI2 (*Δdes*), MS37 (*ΔdesK*), and MS38 (*ΔdesR*) after acute shock with different concentrations of daptomycin (dap) at 37 ºC 18
Figure S13: Growth curves of 168CA (wild type), LAI2 (*Δdes*), MS37 (*ΔdesK*), MS38 (*ΔdesR*) after acute shock with different concentrations of cRRRWFW (cWFW) at 37 ºC 19
Figure S14: Growth curves of 168CA (wild type), LAI2 (*Δdes*), MS37 (*ΔdesK*), MS38 (*ΔdesR*) after acute shock with different concentrations of benzyl alcohol (BA) at 37 ºC 20
Figure S15: Growth curves of 168CA (wild type), LAI2 (*Δdes*), MS37 (*ΔdesK*), MS38 (*ΔdesR*) after acute shock with different concentrations of valinomycin (val) at 25 ºC. 21
Figure S16: Growth curves of 168CA (wild type), LAI2 (*Δdes*), MS37 (*ΔdesK*), MS38 (*ΔdesR*) after acute shock with different concentrations of carbonyl cyanide m-chlorophenylhydrazone (CCCP) at 25 ºC 22
Figure S17: Growth curves of 168CA (wild type), LAI2 (*Δdes*), MS37 (*ΔdesK*), MS38 (*ΔdesR*) after acute shock with different concentrations of vancomycin (van) at 25 ºC 23
Figure S18: Growth curves of 168CA (wild type), LAI2 (*Δdes*), MS37 (*ΔdesK*), MS38 (*ΔdesR*) after acute shock with different concentrations of nisin (nis) at 25 ºC 24
Figure S19: Growth curves of 168CA (wild type), LAI2 (*Δdes*), MS37 (*ΔdesK*), MS38 (*ΔdesR*) after acute shock with different concentrations of daptomycin (dap) at 25 ºC 25
Figure S20: Growth curves of 168CA (wild type), LAI2 (*Δdes*), MS37 (*ΔdesK*), MS38 (*ΔdesR*) after acute shock with different concentrations of cRRRWFW (cWFW) at 25 ºC 26
Figure S21: Growth curves of 168CA (wild type), LAI2 (*Δdes*), MS37 (*ΔdesK*), MS38 (*ΔdesR*) after acute shock with different concentrations of benzyl alcohol (BA) at 25 ºC 27
Figure S22: Membrane fluidity of *B. subtilis* wild type and deletion mutants grown at constant temperature 28
Figure S23: Minimal inhibitory concentrations of daptomycin against *B. subtilis* 168CA (wild type) and the deletion mutant strains LAI2 (*Δdes::ery*) and HB5134 (*Δdes::spc*) at 37 ºC and 24 ºC 29
Figure S24: Percentage of cells with Nile red foci indicative of membrane phase separation 30
Figure S25: PCR confirmation of strains LAI2 (*Δdes::ery*) **(A)**, MS37 (*ΔdesK::ery*), and MS38 (*ΔdesR::ery*) **(B)** using primer pairs MSP101/62 (LAI2) and MSP101/143 (MS37 and MS38) 31
Figure S26: Growth of *B. subtilis* 168CA treated with antibiotics 32

Table S1: Minimal inhibitory concentrations of different antibiotics against *B. subtilis* wild type (WT) and deletion mutants at 37 and 25 °C. Experiments were done in duplicates. Where no standard deviation is given, replicate values were identical.

**antibiotic**	**37 °C**				**25 °C**			
	**168CA (WT)**	**LAI2 (Δ*des::ery*)**	**MS37 (Δ*desK::ery*)**	**MS38 (Δ*desR::ery*)**	**168CA (WT)**	**LAI2 (Δ*des::ery*)**	**MS37 (Δ*desK::ery*)**	**MS38 (Δ*desR::ery*)**
nisin (µg/mL)	6.4	6.4	6.4	3.2	6.4	6.4	6.4	6.4
vancomycin (µg/mL)	0.25	0.25	0.25	0.25	0.125	0.125	0.125	0.25
cWFW (µg/mL)	32	32	32	32	32	32	32	32
CCCP (µM)	6.25	6.25	6.25	6.25	6.25	6.25	3.125	6.25
benzyl alcohol (mM)	50	50	50	50	50	50	50	50
daptomycin (µg/mL)	2	2	2	2	1	1	1	1
valinomycin (µg/mL)	2.25 ± 0.6	2.7 ± 0.6	1	2.5 ± 0.7	1	1	1	1

Table S2: Strains and plasmids used in this work.

strain	genotype	origin[a]
E. coli		
DH5α	*fhuA2 lacΔU169 phoA glnV44 Φ80' lacZΔM15 gyrA96 recA1 relA1 endA1 thi-1 hsdR17*	NEB
TOP10	*F–mcrA Δ(mrr-hsdRMS-mcrBC) φ80lacZΔM15 ΔlacX74 recA1 araD139 Δ(ara-leu)7697 galU galK λ–rpsL(StrR) endA1 nupG*	ThermoFisher
B. subtilis		
168CA	*trpC2*	(1)
AKP3	*trpC2 pheA1 amyE::cat Pdes-lacZ*	(2)
AM1	*trpC2 amyE::spc Pxyl-desK-msfgfp*	this work<br>pAM1 →168CA
BKE19180	*trpC2 Δdes::ery*	(3)
BKE19190	*trpC2 ΔdesK::ery*	(3)
BKE19200	*trpC2 ΔdesR::ery*	(3)
HB5134	*trpC2 Δdes::spc*	(4)
JH642	*trpC2 pheA1*	(2)
LAI2	*trpC2 Δdes::ery*	this work<br>BKE19180 →168CA
MS37	*trpC2 ΔdesK::ery*	this work<br>BKE19190 →168CA
MS38	*trpC2 ΔdesR::ery*	this work<br>BKE19200→168CA
MS46	*trpC2 amyE::cat Pdes-lacZ*	this work<br>AKP3 →168CA
Plasmids		
pAM1	*bla amyE::spc Pxyl-desK-msfgfp*	this work
pMW1	*bla amyE::spc Pxyl-msfgfp*	(5)

[a]Arrow indicates transformation of the plasmid or chromosomal DNA into the respective strain (DNA→ Strain).

Table S3: Primers used in this work.

DNA template	primer	primer sequence[a]	PCR product
Gibson assembly: vector backbone linearization			
pMW1	MWP1	ATGAGCAAAGGAGAAGAACTTTTC	Linear pMW1
	Abs1	CCTAGGAATCTCCTTTCTAGATGC	
Gibson assembly: insert amplification			
168CA	MSP11	CTAGAAAGGAGATTCCTAGGATGATTAAAAATCAT TTTACATTTCAAAAACTAAACGGG	*desK* (insert)
	MSP93	AGTTCTTCTCCTTTGCTCATGCTTCCGCTTCCGCTTC CGCTTCCGCTTCCGCTTCCTTTTGAATTATTAGGAA TTGCCATGGTAAGC	
Plasmid and strain confirmation			
pAM1	TerS21	GGGCAACAAACTAATGTGCAA	1298 bp
	Abs5	GAAAATTTGTGCCCATTAACATCACCATC	
LAI2	MSP101	GCTCATTGGCATTACTTTTAATGGC	1104 bp
	MSP62	AGAGAAAGCTTTTGCCCAAGC	
MS37, MSP38	MSP101	GCTCATTGGCATTACTTTTAATGGC	1205 bp (MS37); 587 bp (MS38)
	MSP143	CATTCAATCCAGTCTTTTATTGGGCTG	
AM1, MS46	Absp11	GTGATAATTTTAAATGTAAGCGTTAACAAAATTC	5191 bp (AM1); 7000 bp (MS46)
	Absp12	CTGATTTATGAACAAAAAAGAAACCATCATTG	

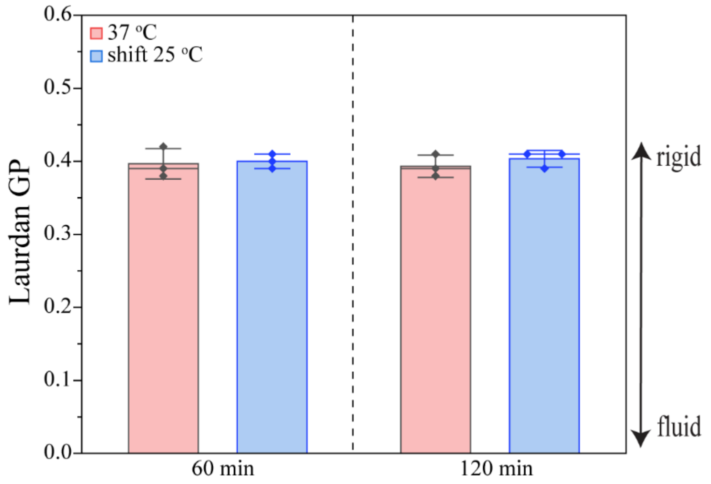

Figure S1: Membrane fluidity measurements using laurdan generalized polarization (GP) spectroscopy. An increase in GP indicates membrane rigidification, a decrease fluidization. *B. subtilis* 168CA was grown at 37 °C until early log phase (OD_{600}=0.3) prior to shifting to 25 ºC.

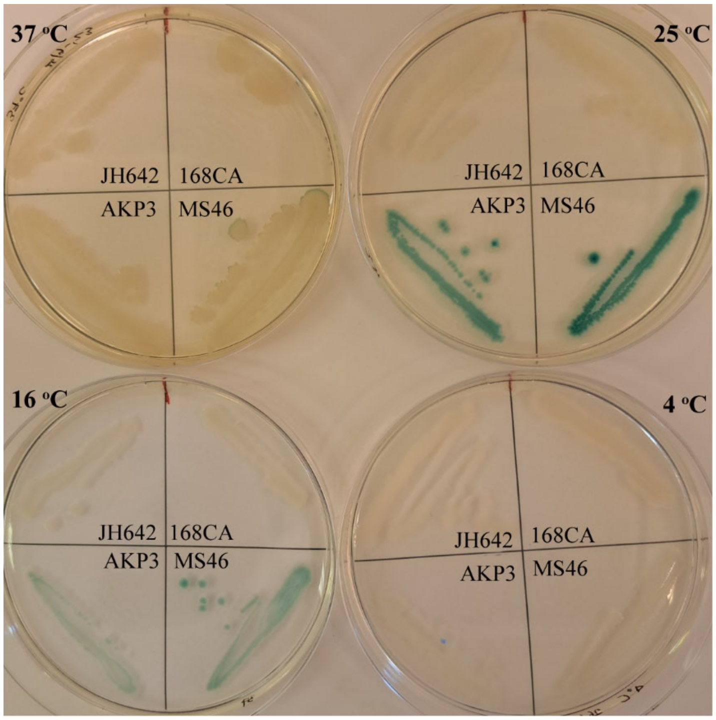

Figure S2: *Pdes* promotor activation measured by β-galactosidase activity on agar plates supplemented with X-gal. *B. subtilis* strains were grown at 37 ºC overnight and subsequently shifted to 25, 16, and 4 ºC, respectively. Pictures were taken 3 days after temperature shift. AKP3 (*trpC2 pheA2 amyE::cat Pdes-lacZ*): original strain from Aguilar *et al*.(2), JH642 (*trpC2 pheA2*): WT background of AKP3, MS46 (*trpC2 amyE::cat Pdes-lacZ*): strain used in this study (AKP3 → 168CA), 168CA (*trpC2*): WT background of MS46.

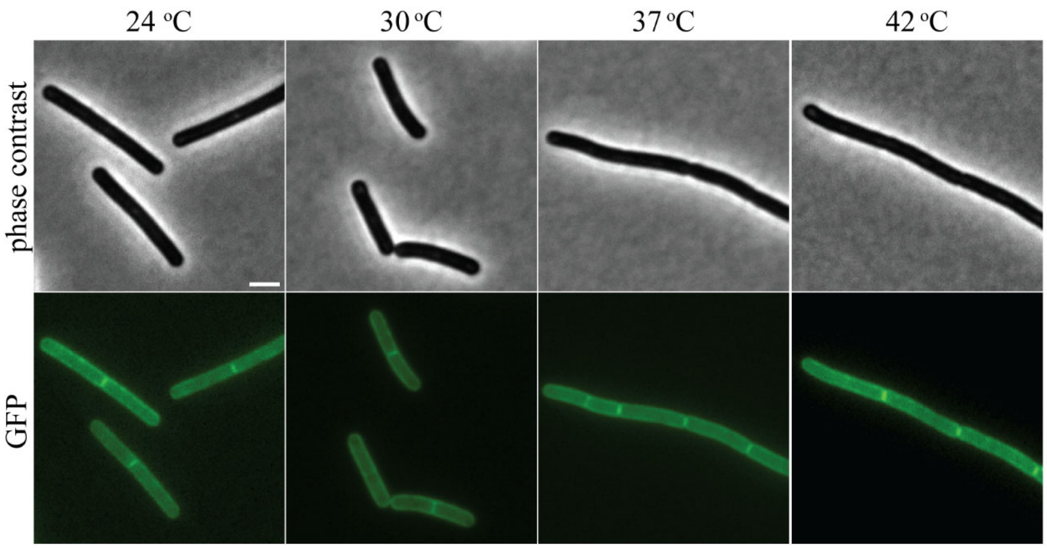

Figure S3: Localization of DesK-GFP at different permissive temperatures. *B. subtilis* MS46 (*Pxyl-desK-msfgfp*) was grown at the indicated temperatures until early log phase (OD_{600}=0.3) prior to microscopy. It was not possible to obtain images of cells grown at 4, 16, or 50 °C as cultures did not grow under these conditions. Scale bar represents 2 μm.

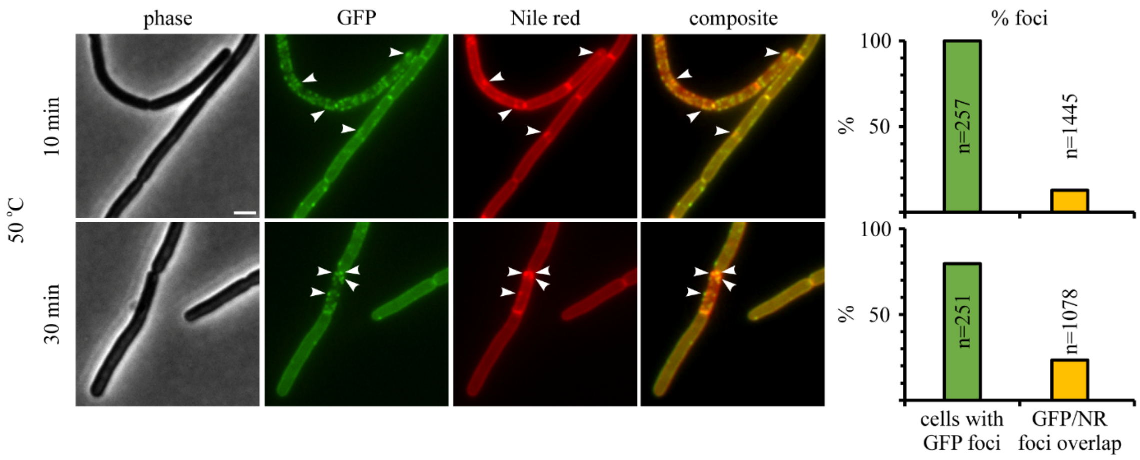

Figure S4: Localization of DesK after temperature shift to 50 °C. *B. subtilis* AM1 (*Pxyl-desK-msfgfp*) was grown at 37 °C until an OD_{600} of 0.3 and subsequently shifted to 50 °C. Cells were stained with Nile red for 5 min prior to microscopy. Scale bar represents 2 µm. Arrows indicate GFP clusters overlapping with Nile red foci. Cells from three replicate experiments were pooled for quantification. n indicates the total number of counted cells or GFP foci, respectively.

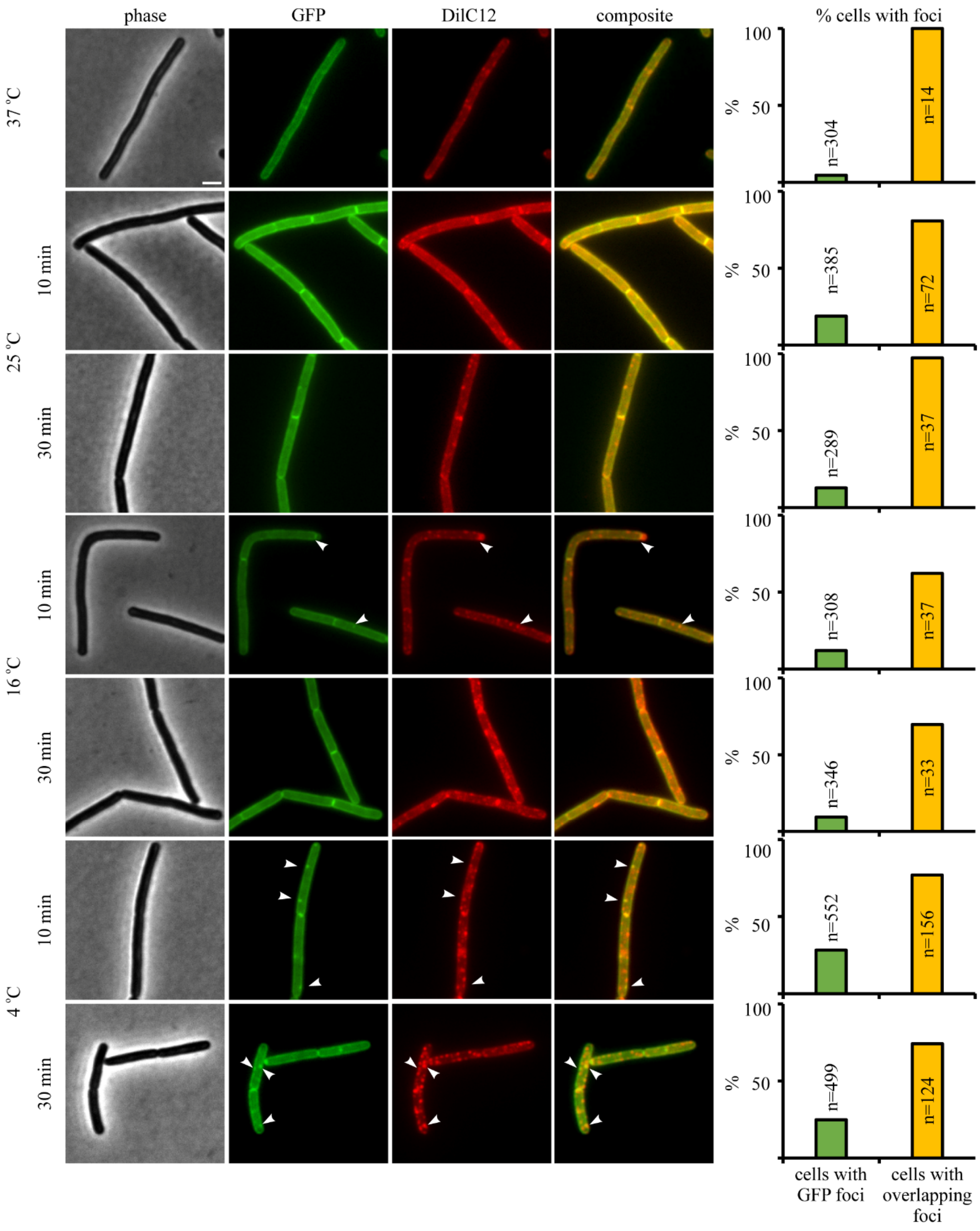

Figure S5: Co-localization of DesK with DiIC12 after temperature shock. *B. subtilis* AM1 (*Pxyl-desK-msfgfp*) was grown at 37 °C until an OD_{600} of 0.3 and subsequently shifted to the indicated temperatures. Scale bar represents 2 µm. Arrows indicate GFP clusters overlapping with DiIC12 foci. Cells from three replicate experiments were pooled for quantification. n indicates the total number of counted cells.

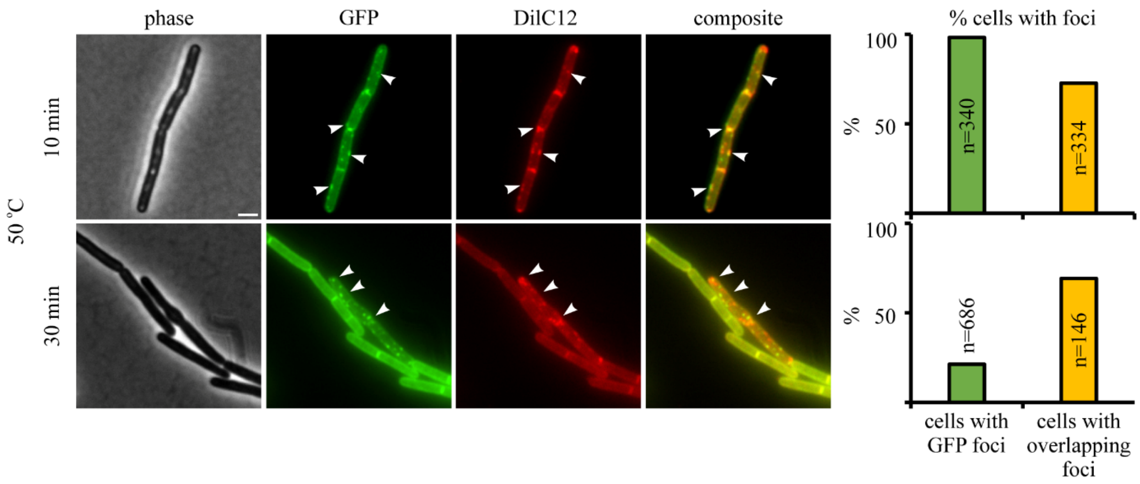

Figure S6: Co-localization of DesK with DiIC12 after temperature shock to 50 °C. *B. subtilis* AM1 (*Pxyl-desK-msfgfp*) was grown at 37 °C until an OD_{600} of 0.3 and subsequently shifted to 50 °C. Scale bar represents 2 µm. Arrows indicate GFP clusters overlapping with DiIC12 foci. Cells from three replicate experiments were pooled for quantification. n indicates the total number of counted cells.

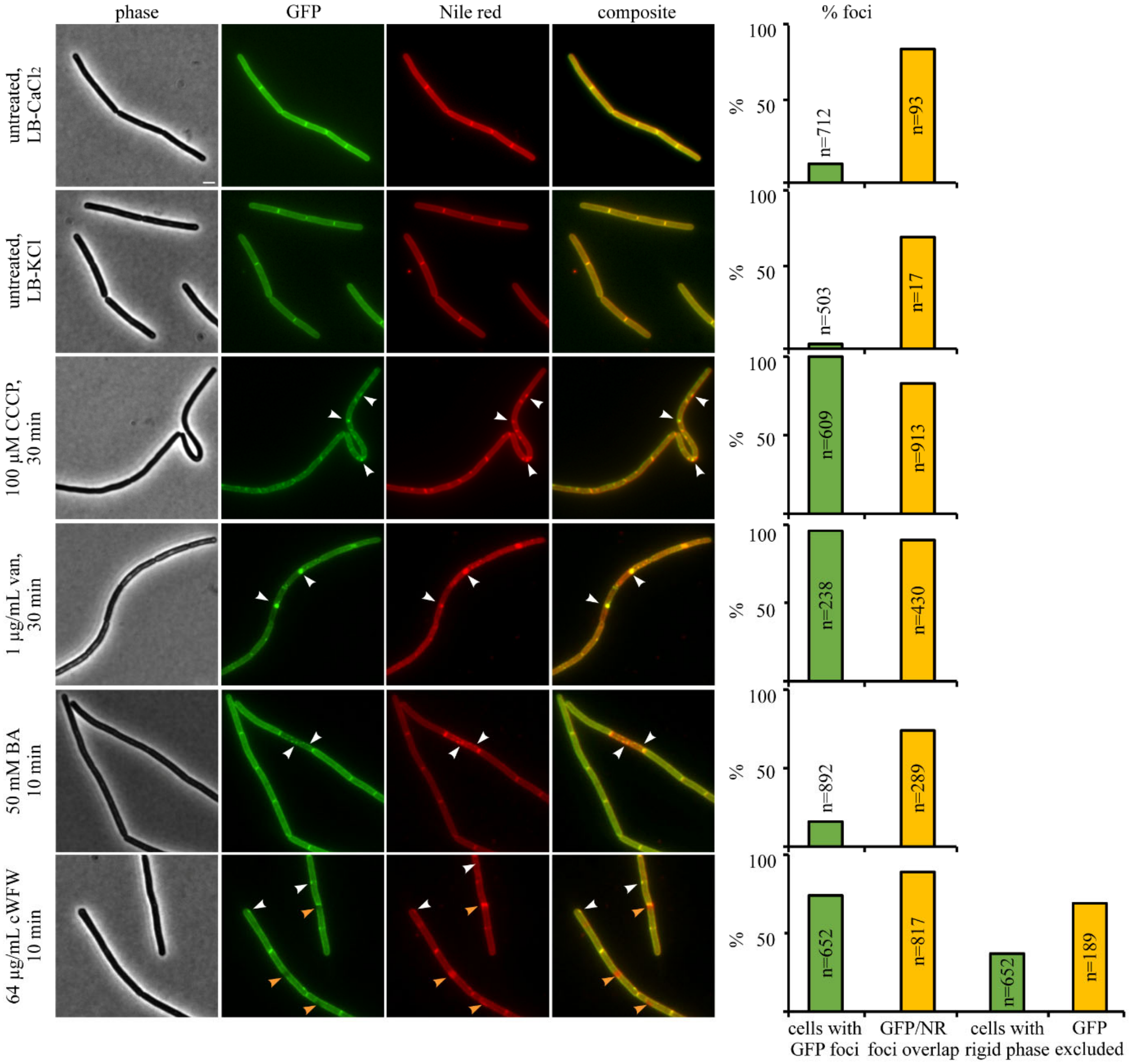

Figure S7: Localization of DesK after antibiotic treatment (additional controls and timepoints). *B. subtilis* AM1 (*Pxyl-desK-msfgfp*) was grown at 37 °C until an OD_{600} of 0.3 and subsequently treated with vancomycin (van), CCCP, cRRRWFW (cWFW), and benzyl alcohol (BA), followed by staining with Nile red. White arrows indicate GFP clusters overlapping with Nile red foci. Orange arrows mark rigid domains that are void of DesK-GFP but stained by Nile red. Cells from three replicate experiments were pooled for quantification. n indicates the total number of counted cells and GFP foci, respectively.

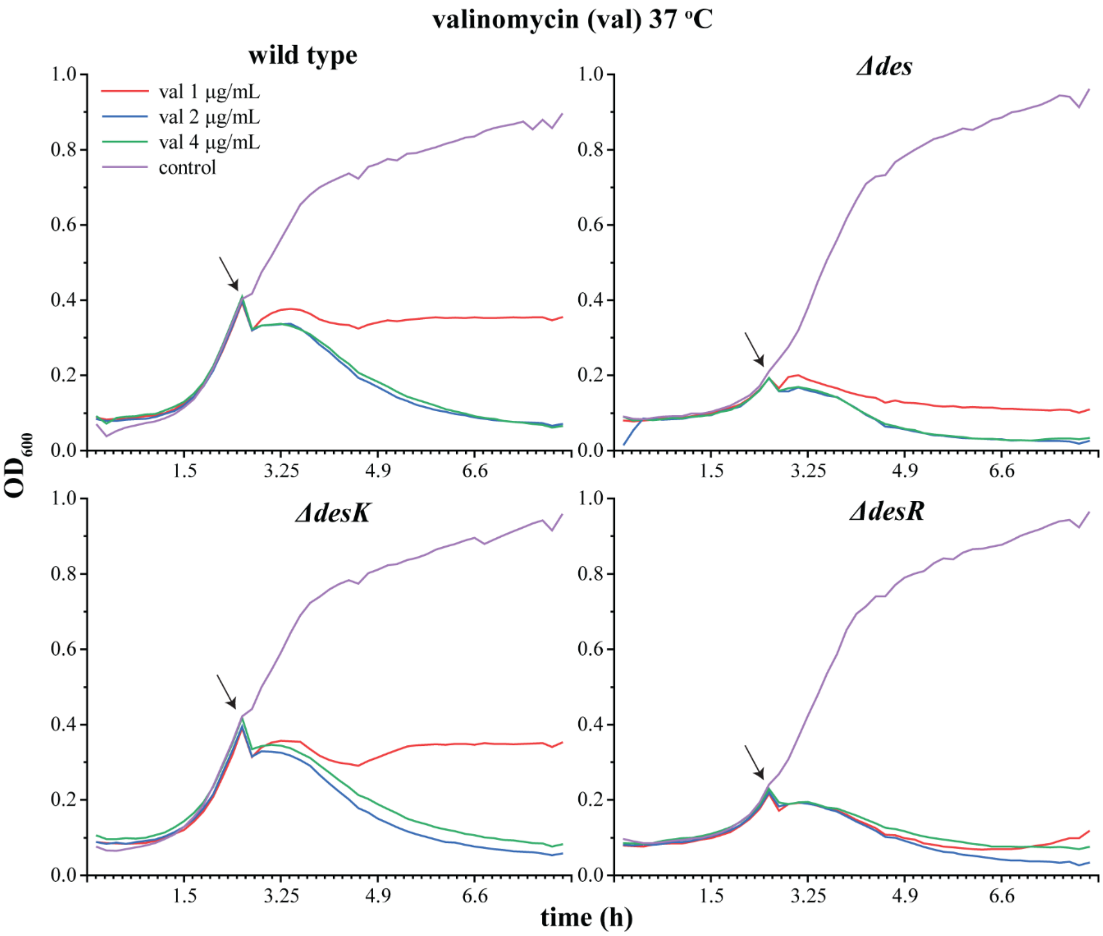

Figure S8: Growth curves of 168CA (wild type), LAI2 (*Δdes*), MS37 (*ΔdesK*), MS38 (*ΔdesR*) after acute shock with different concentrations of valinomycin (val) at 37 ºC. Arrows indicate timepoints of antibiotic addition. All cultures were grown in tryptone-yeast (TY) medium containing 300 mM KCl.

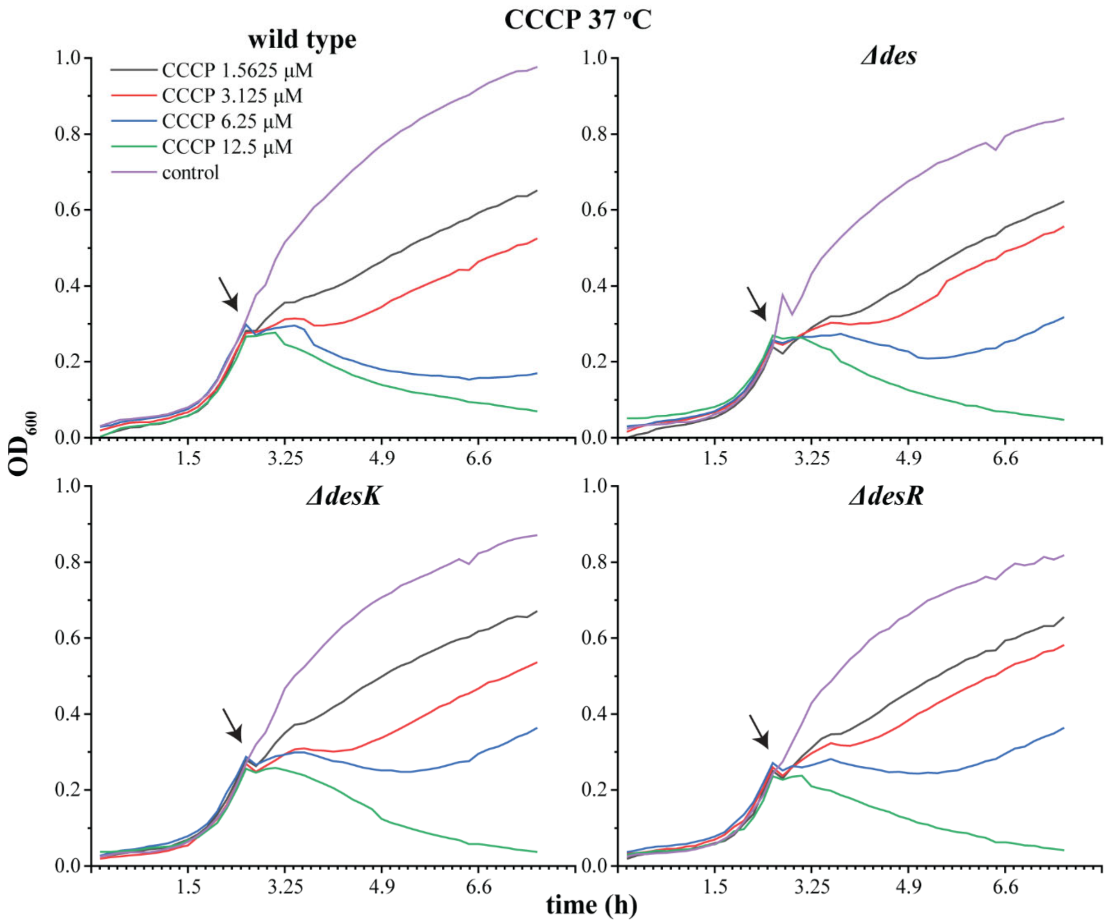

Figure S9: Growth curves of 168CA (wild type), LAI2 (*Δdes*), MS37 (*ΔdesK*), MS38 (*ΔdesR*) after acute shock with different concentrations of carbonyl cyanide m-chlorophenylhydrazone (CCCP) at 37 °C. Arrows indicate timepoints of antibiotic addition.

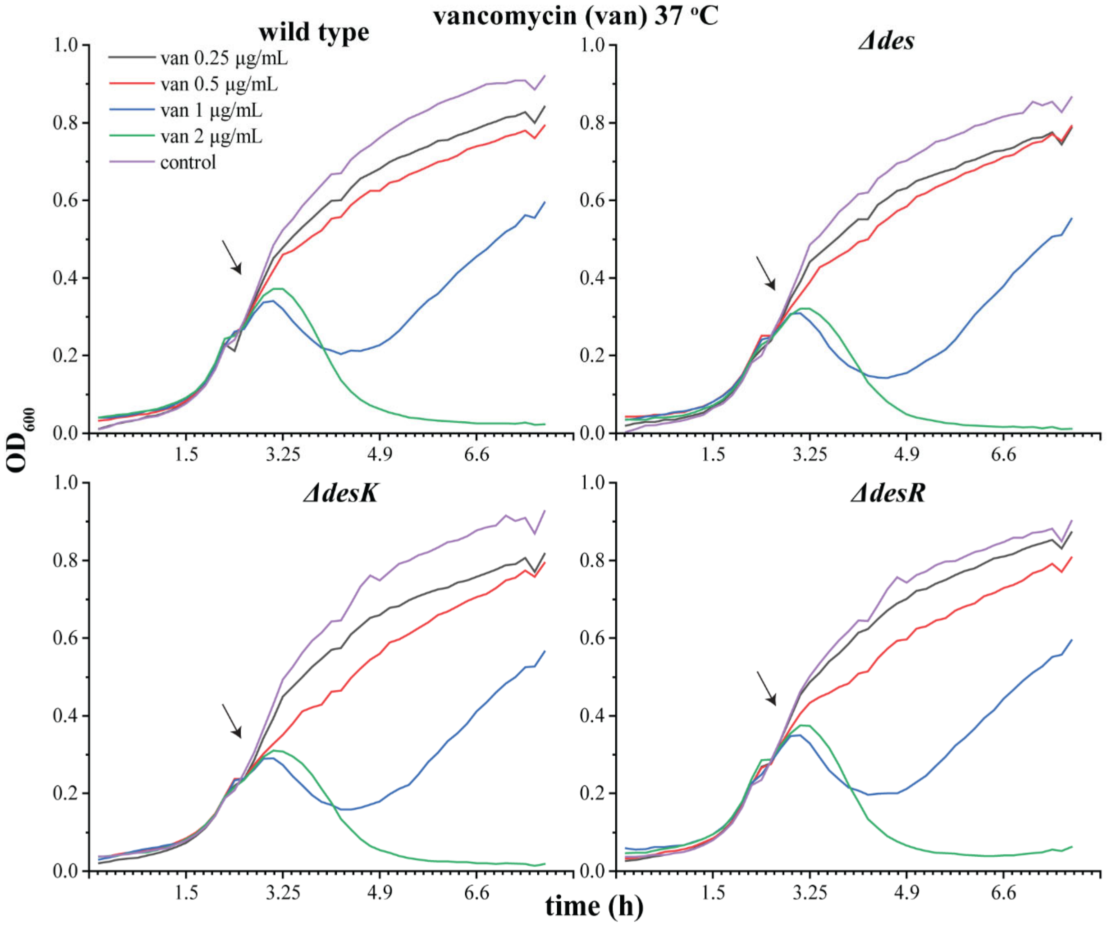

Figure S10: Growth curves of 168CA (wild type), LAI2 (*Δdes*), MS37 (*ΔdesK*), MS38 (*ΔdesR*) after acute shock with different concentrations of vancomycin (van) at 37 ºC. Arrows indicate timepoints of antibiotic addition.

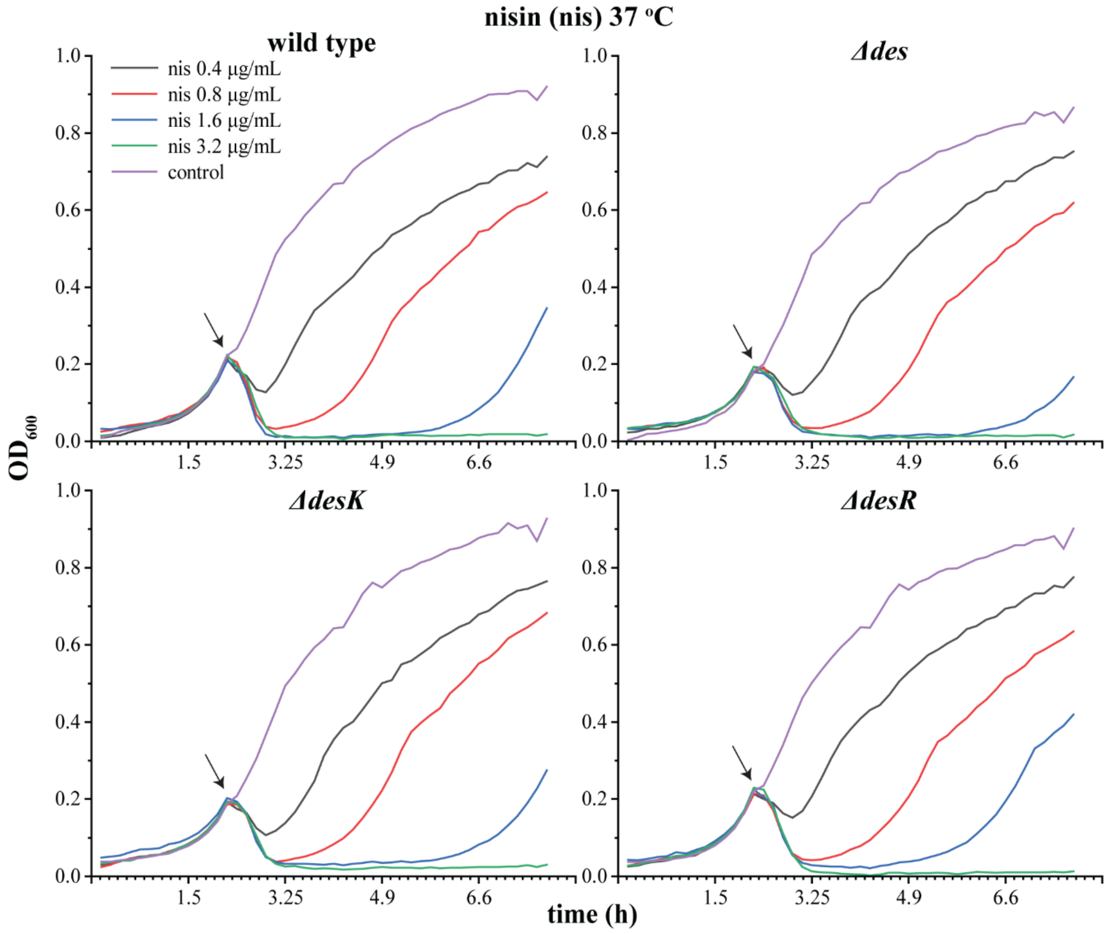

Figure S11: Growth curves of 168CA (wild type), LAI2 (*Δdes*), MS37 (*ΔdesK*), MS38 (*ΔdesR*) after acute shock with different concentrations of nisin (nis) at 37 ºC. Arrows indicate timepoints of antibiotic addition.

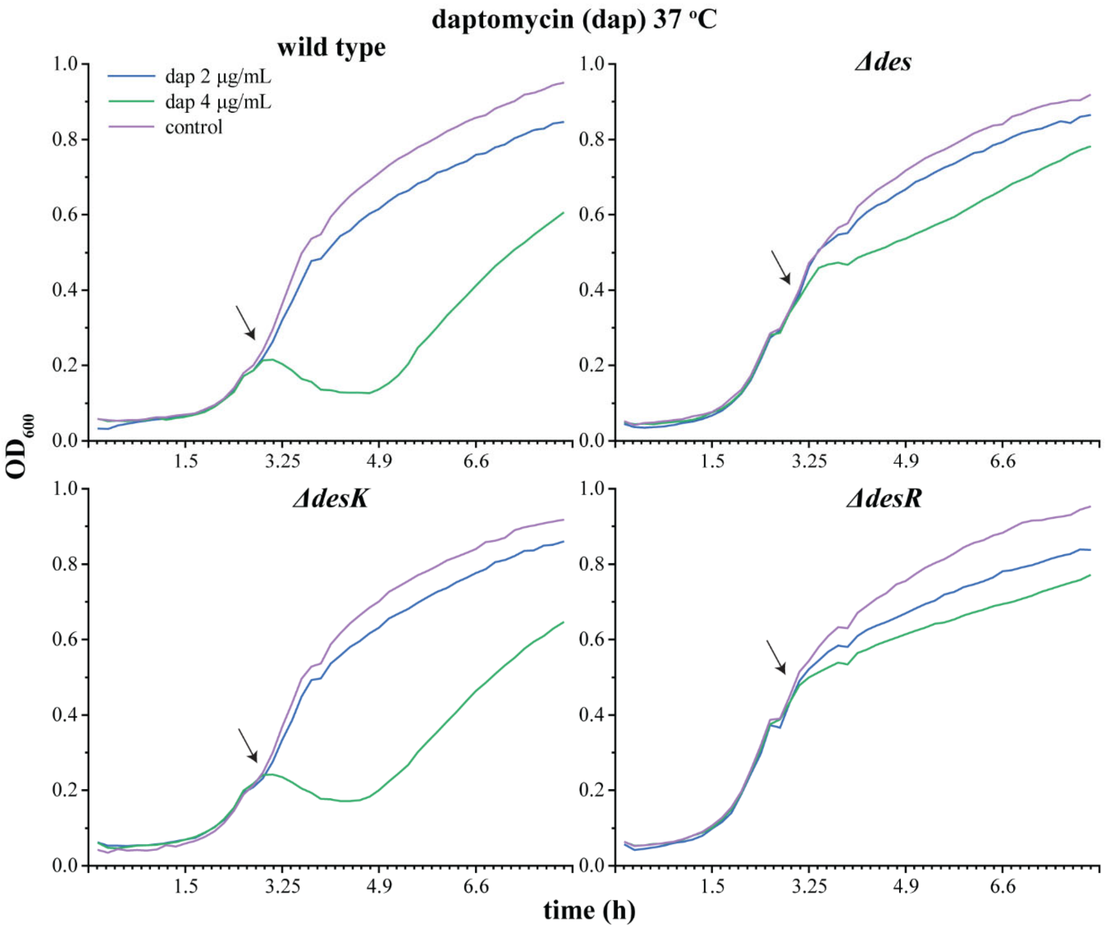

Figure S12: Growth curves of 168CA (wild type), LAI2 (*Δdes*), MS37 (*ΔdesK*), and MS38 (*ΔdesR*) after acute shock with different concentrations of daptomycin (dap) at 37 ºC. Arrows indicate timepoints of antibiotic addition. All cultures were grown in the presence of 1.25 mM $CaCl_2$.

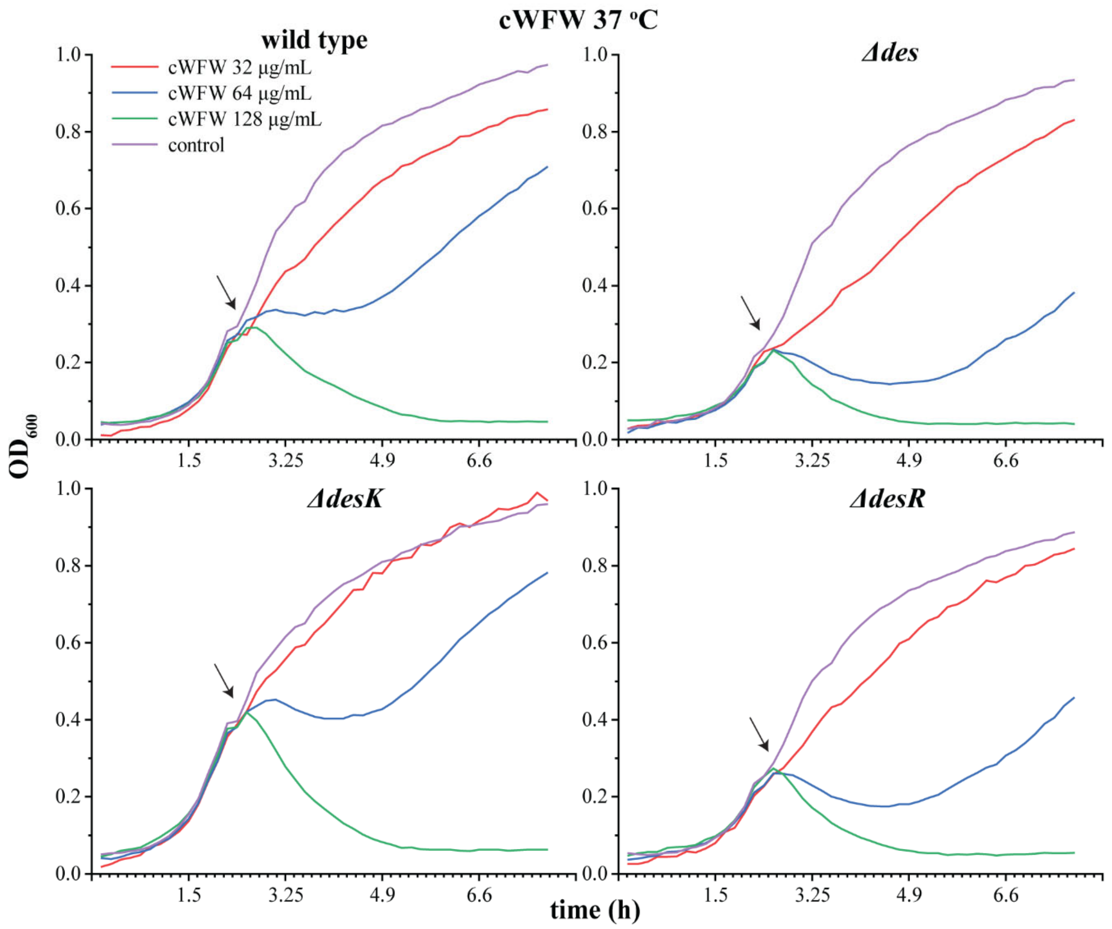

Figure S13: Growth curves of 168CA (wild type), LAI2 (*Δdes*), MS37 (*ΔdesK*), MS38 (*ΔdesR*) after acute shock with different concentrations of cRRRWFW (cWFW) at 37 ºC. Arrows indicate timepoints of antibiotic addition.

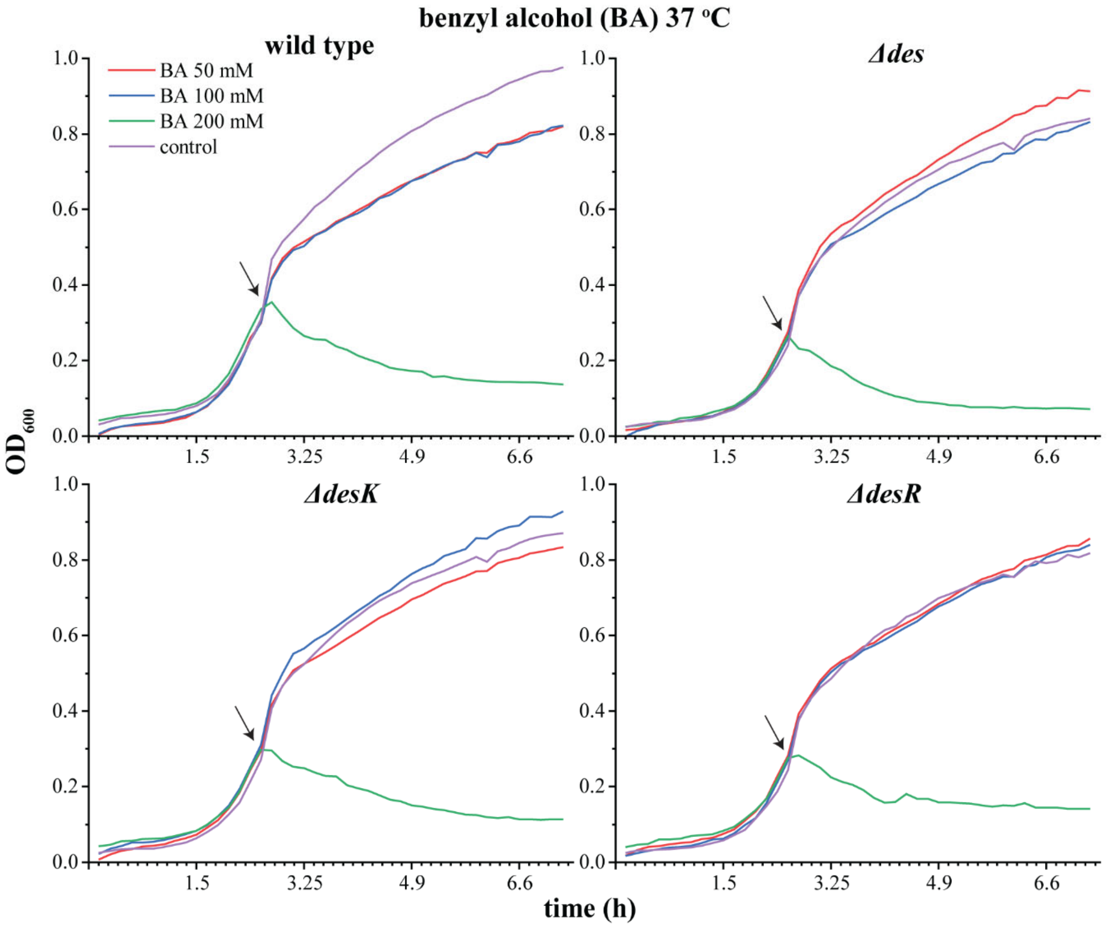

Figure S14: Growth curves of 168CA (wild type), LAI2 (*Δdes*), MS37 (*ΔdesK*), MS38 (*ΔdesR*) after acute shock with different concentrations of benzyl alcohol (BA) at 37 ºC. Arrows indicate timepoints of antibiotic addition.

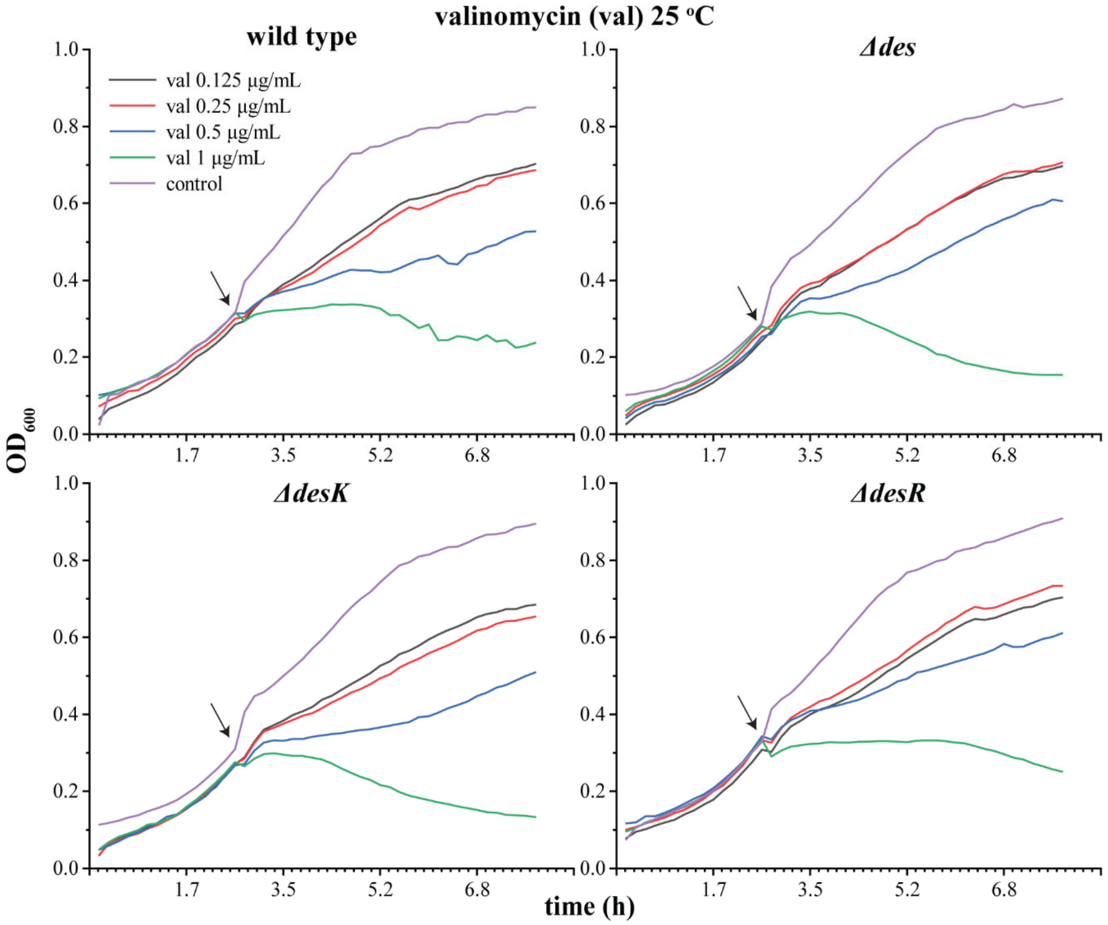

Figure S15: Growth curves of 168CA (wild type), LAI2 (*Δdes*), MS37 (*ΔdesK*), MS38 (*ΔdesR*) after acute shock with different concentrations of valinomycin (val) at 25 ºC. Arrows indicate timepoints of antibiotic addition. All cultures were grown in tryptone-yeast (TY) medium containing 300 mM KCl.

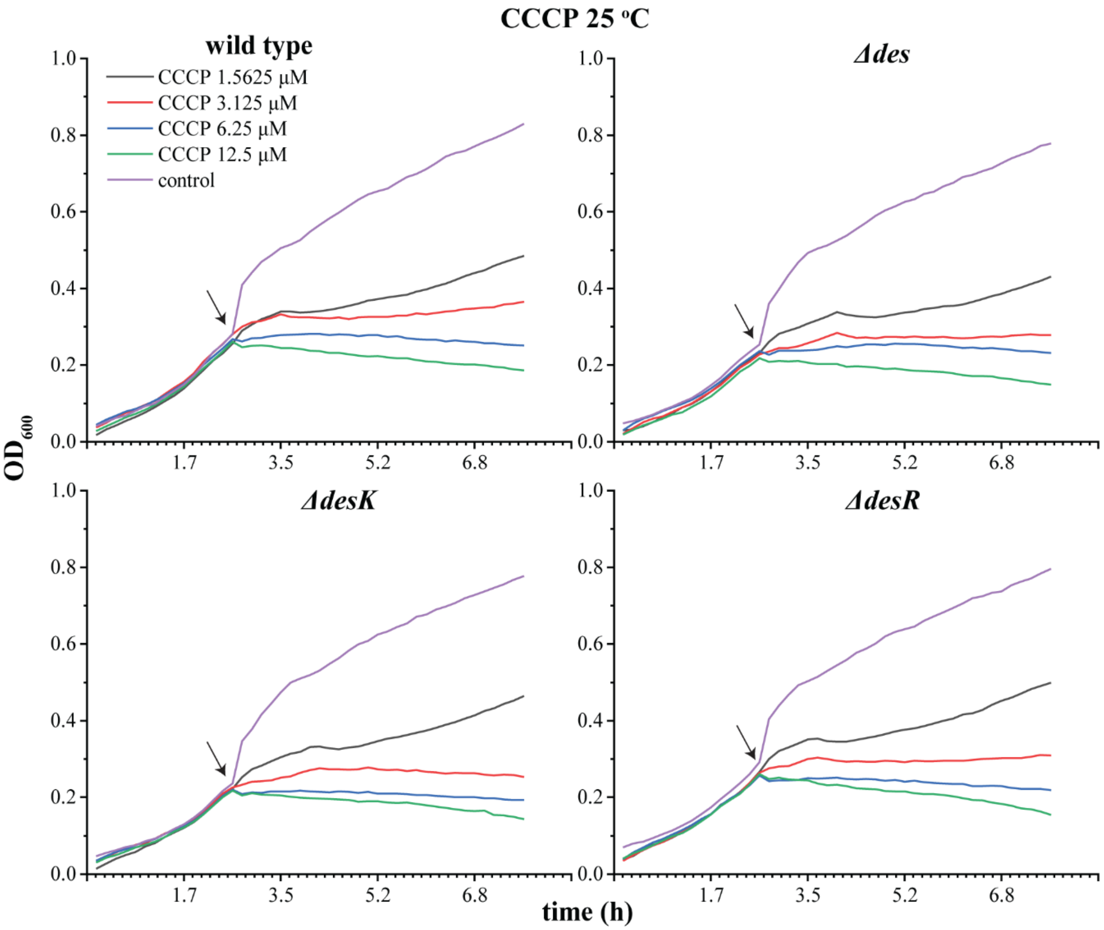

Figure S16: Growth curves of 168CA (wild type), LAI2 (*Δdes*), MS37 (*ΔdesK*), MS38 (*ΔdesR*) after acute shock with different concentrations of carbonyl cyanide m-chlorophenylhydrazone (CCCP) at 25 ºC. Arrows indicate timepoints of antibiotic addition.

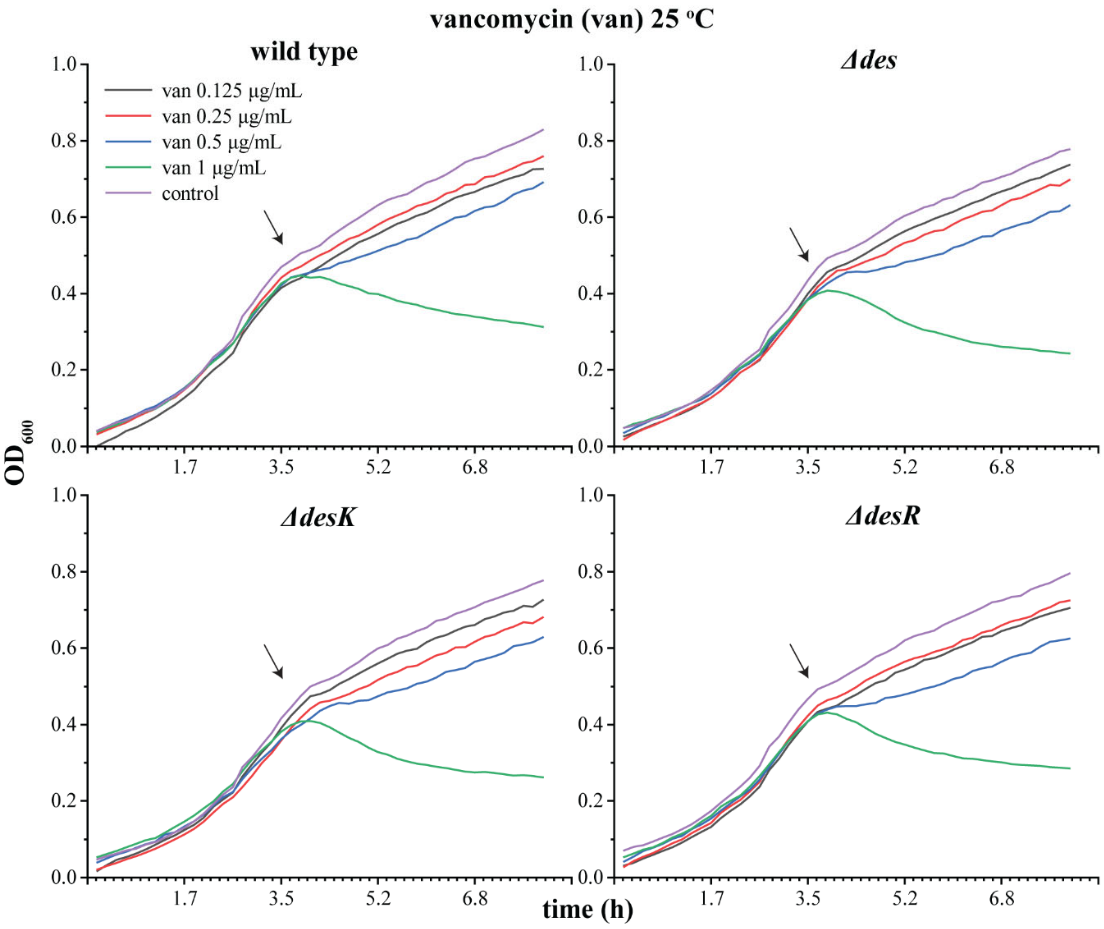

Figure S17: Growth curves of 168CA (wild type), LAI2 (*Δdes*), MS37 (*ΔdesK*), MS38 (*ΔdesR*) after acute shock with different concentrations of vancomycin (van) at 25 °C. Arrows indicate timepoints of antibiotic addition.

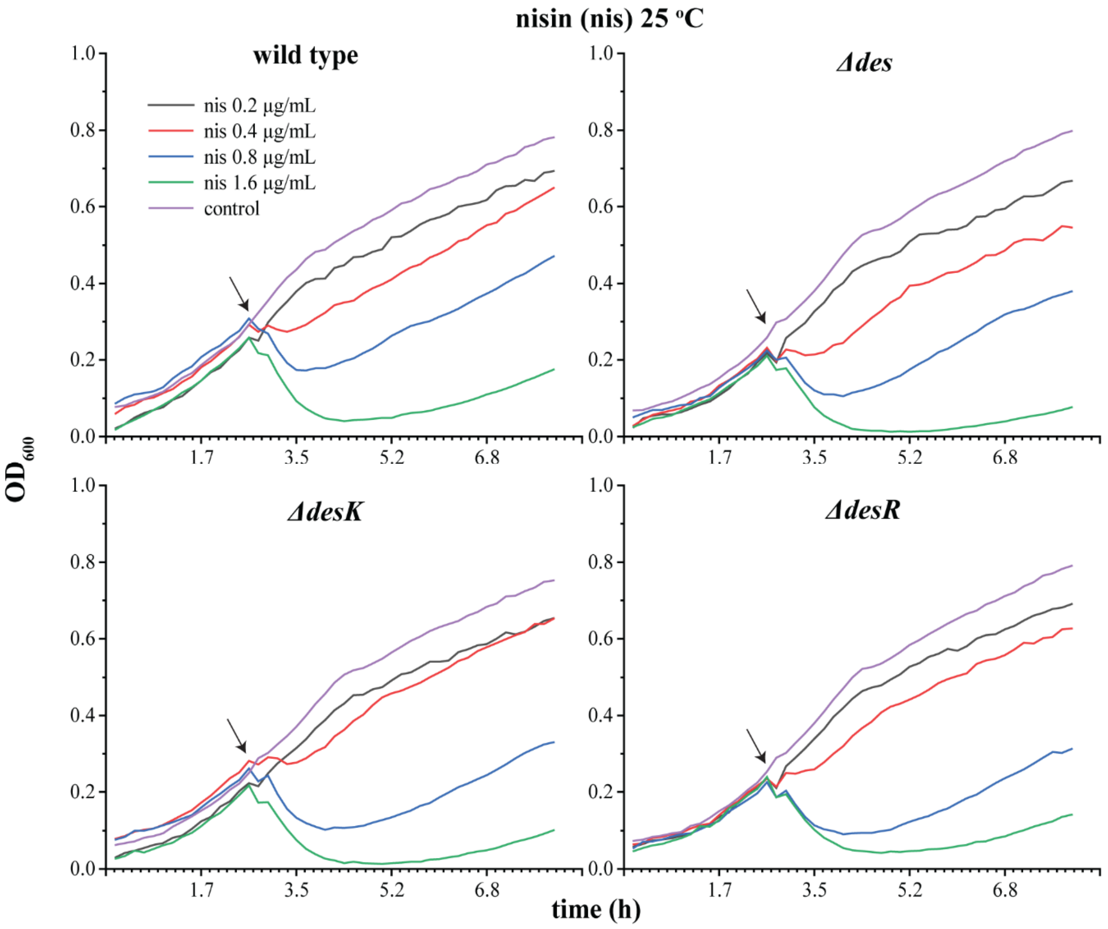

Figure S18: Growth curves of 168CA (wild type), LAI2 (*Δdes*), MS37 (*ΔdesK*), MS38 (*ΔdesR*) after acute shock with different concentrations of nisin (nis) at 25 ºC. Arrows indicate timepoints of antibiotic addition.

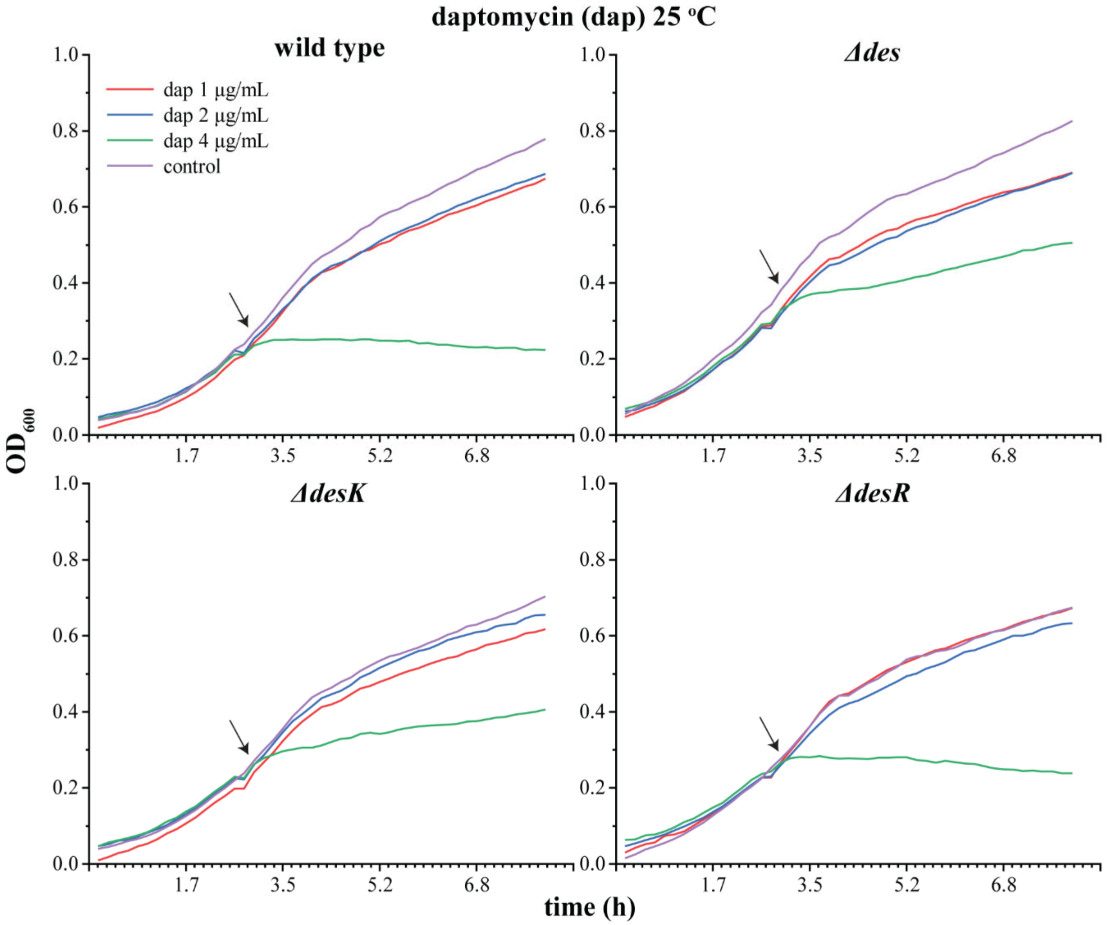

Figure S19: Growth curves of 168CA (wild type), LAI2 (*Δdes*), MS37 (*ΔdesK*), MS38 (*ΔdesR*) after acute shock with different concentrations of daptomycin (dap) at 25 ºC. Arrows indicate timepoints of antibiotic addition. All cultures were grown in the presence of 1.25 mM $CaCl_2$.

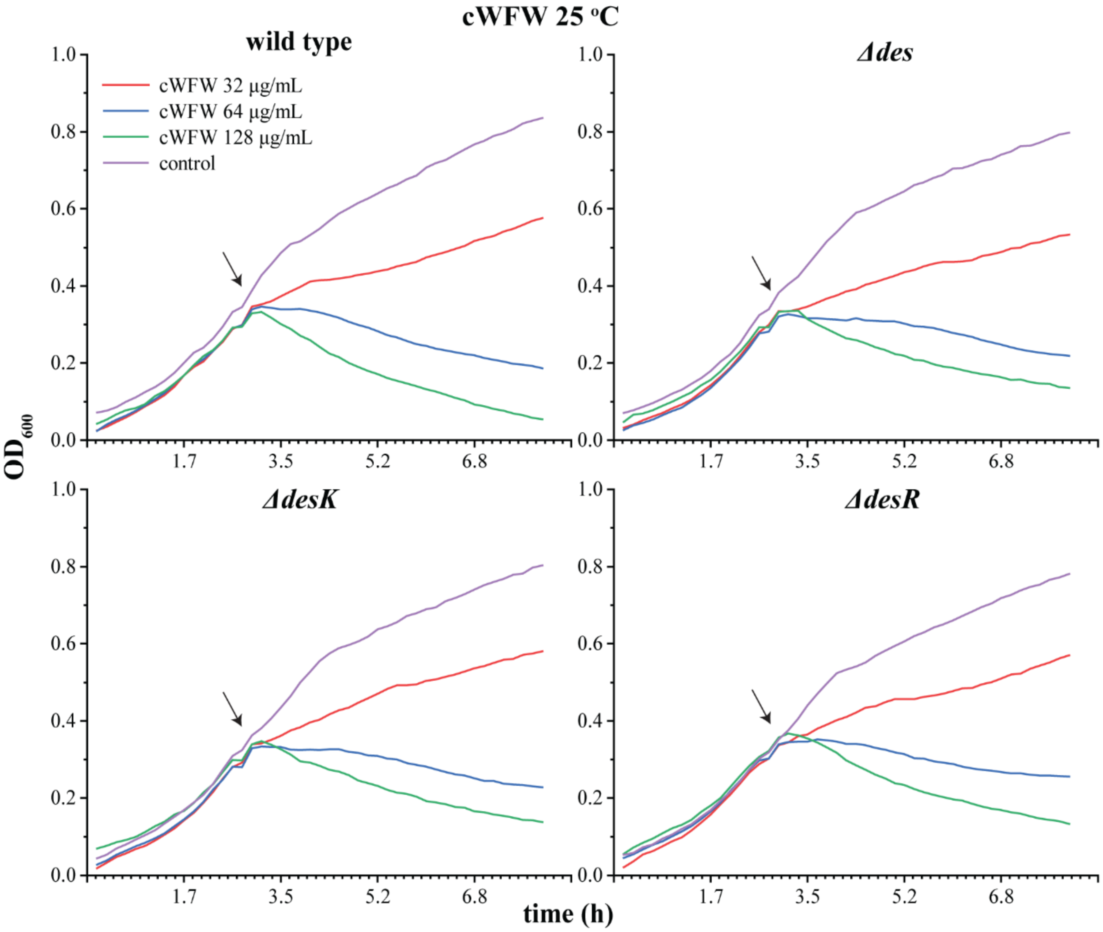

Figure S20: Growth curves of 168CA (wild type), LAI2 (*Δdes*), MS37 (*ΔdesK*), MS38 (*ΔdesR*) after acute shock with different concentrations of cRRRWFW (cWFW) at 25 ºC. Arrows indicate timepoints of antibiotic addition.

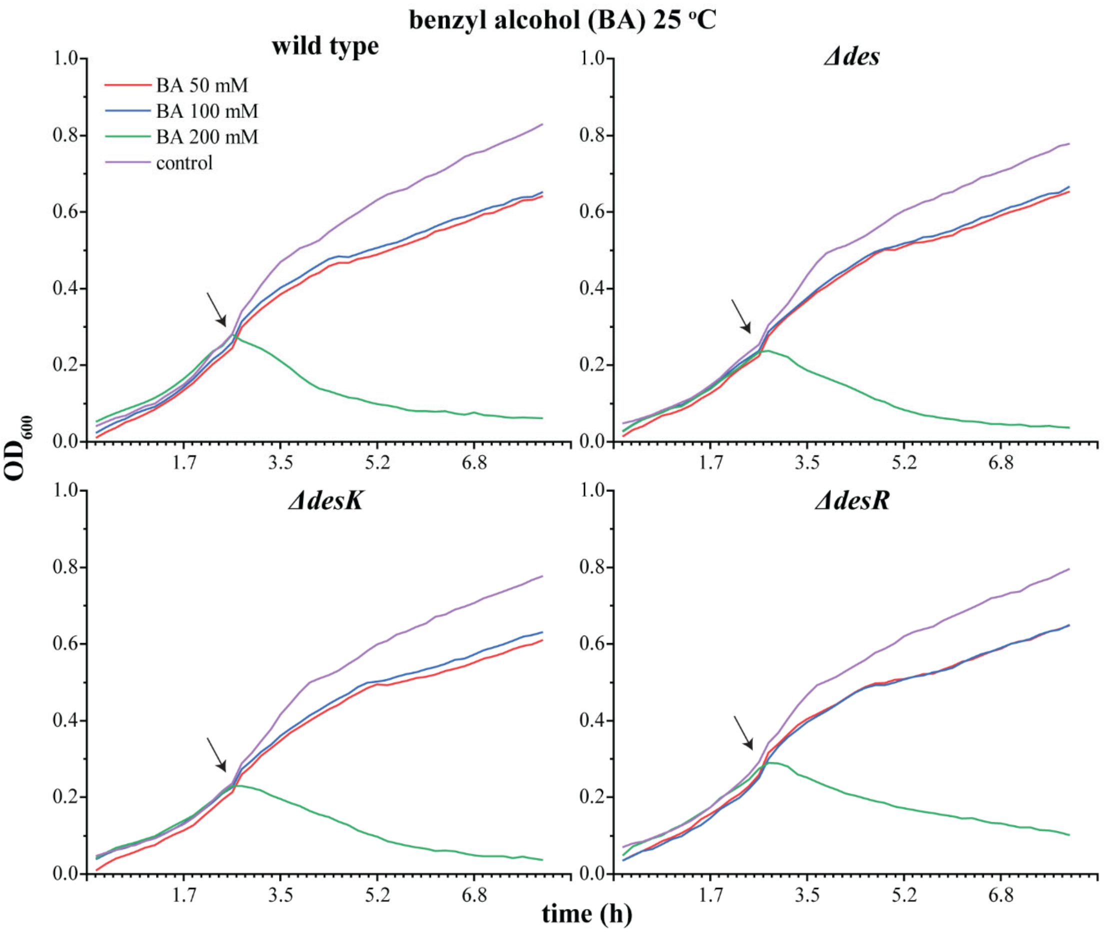

Figure S21: Growth curves of 168CA (wild type), LAI2 (*Δdes*), MS37 (*ΔdesK*), MS38 (*ΔdesR*) after acute shock with different concentrations of benzyl alcohol (BA) at 25 °C. Arrows indicate timepoints of antibiotic addition.

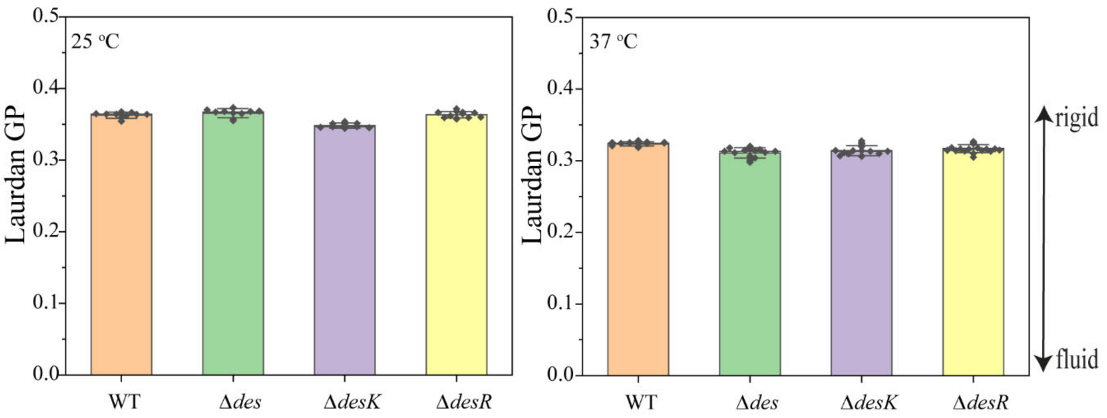

Figure S22: Membrane fluidity of *B. subtilis* wild type and deletion mutants grown at constant temperature. *B. subtilis* 168CA (wild type), LAI2 (*Δdes::ery*), MS37 (*ΔdesK::ery*), MS38 (*ΔdesR::ery*) were grown at either 25 or 37 °C until log phase prior to fluidity measurements. Error bars represent standard deviation of the mean. Statistical significance was determined using two-tailed, homoscedastic t-tests but no significant ($p \leq 0.05$) differences were observed.

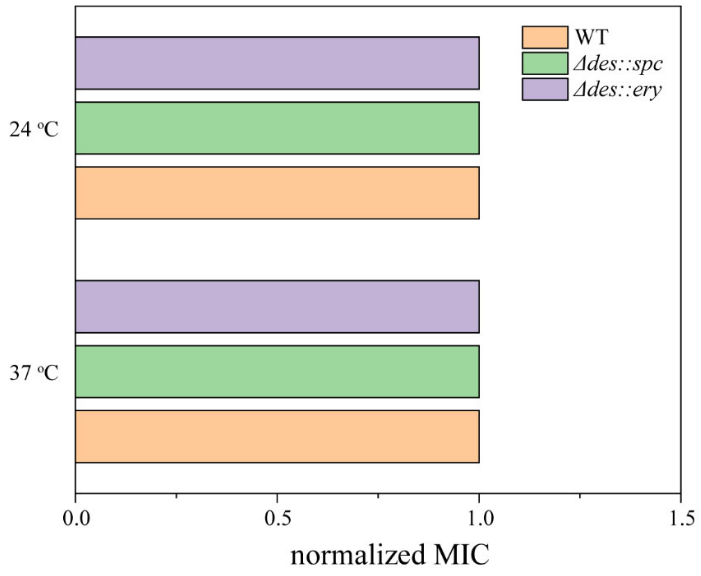

Figure S23: Minimal inhibitory concentrations of daptomycin against *B. subtilis* 168CA (wild type) and the deletion mutant strains LAI2 (*Δdes::ery*) and HB5134 (*Δdes::spc*) at 37 ºC and 24 ºC. Values are expressed relative to the wild type MIC (1 µg/mL for 37 °C and 0.5 µg/mL for 24 °C). No error bars are shown as replicate values of four biological replicates were identical.

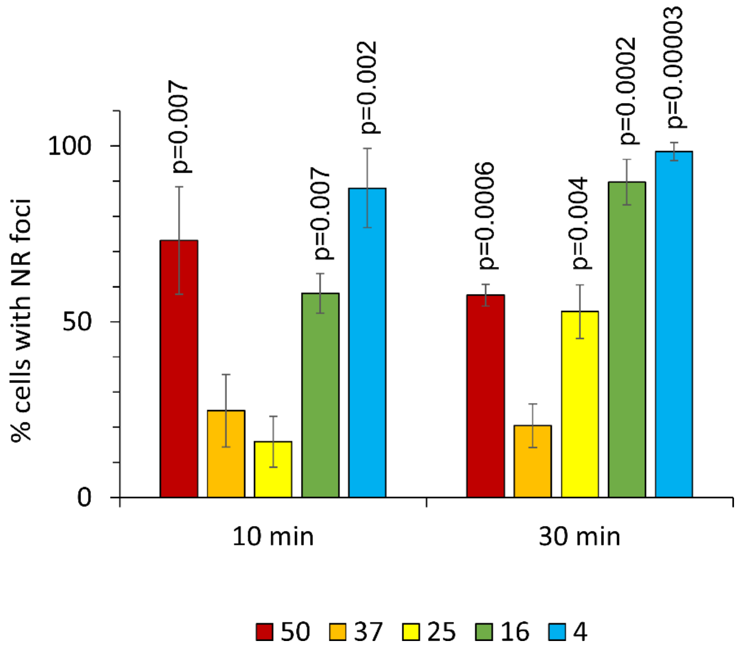

Figure S24: Percentage of cells with Nile red foci indicative of membrane phase separation. *B. subtilis* AM1 (*Pxyl-desK-msfgfp*) was grown at 37 °C until an OD_{600} of 0.3 and subsequently shifted to the indicated temperatures. Cells were stained with Nile red for 5 min prior to microscopy. Error bars represent standard deviation of the mean. Statistical significance was determined using two-tailed, homoscedastic t-tests. Only p values ≤0.05 are indicated.

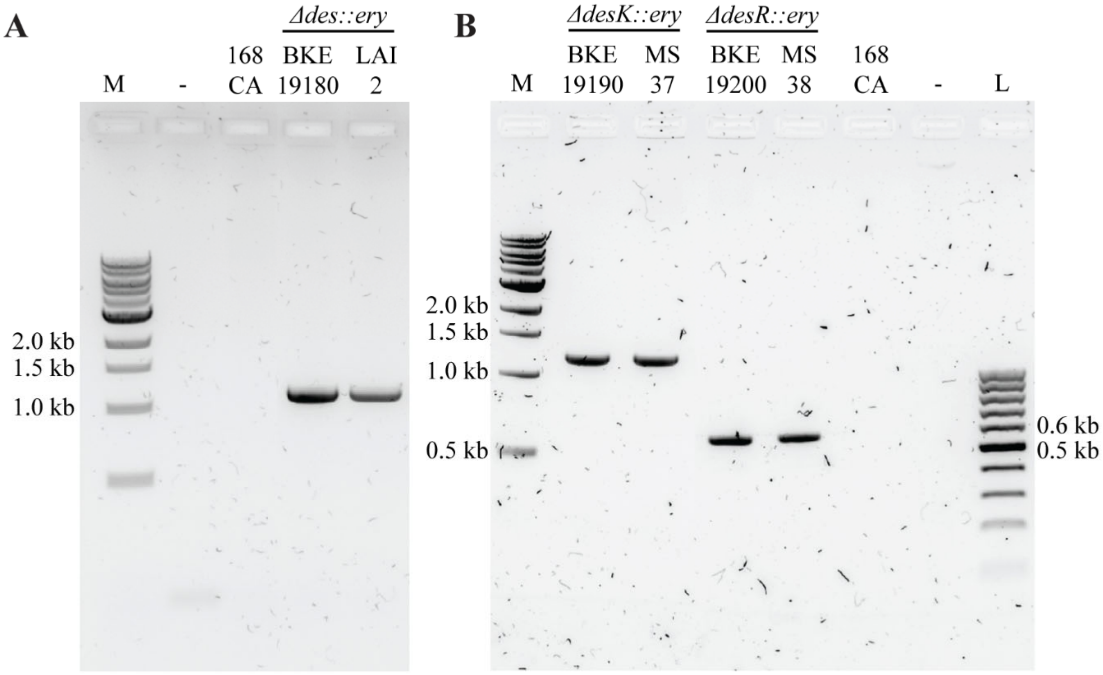

Figure S25: PCR confirmation of strains LAI2 (*Δdes::ery*) **(A)**, MS37 (*ΔdesK::ery*), and MS38 (*ΔdesR::ery*) **(B)** using primer pairs MSP101/62 (LAI2) and MSP101/143 (MS37 and MS38). Expected PCR products for LAI2, MS37, and MS38 were 1104 bp, 1205 bp, and 587 bp, respectively. M and L refer to NEB 1 kb DNA ladder and Thermo Scientific GeneRuler 100 bp DNA ladder, respectively. The negative control (-) contained only PCR master mix and water without DNA.

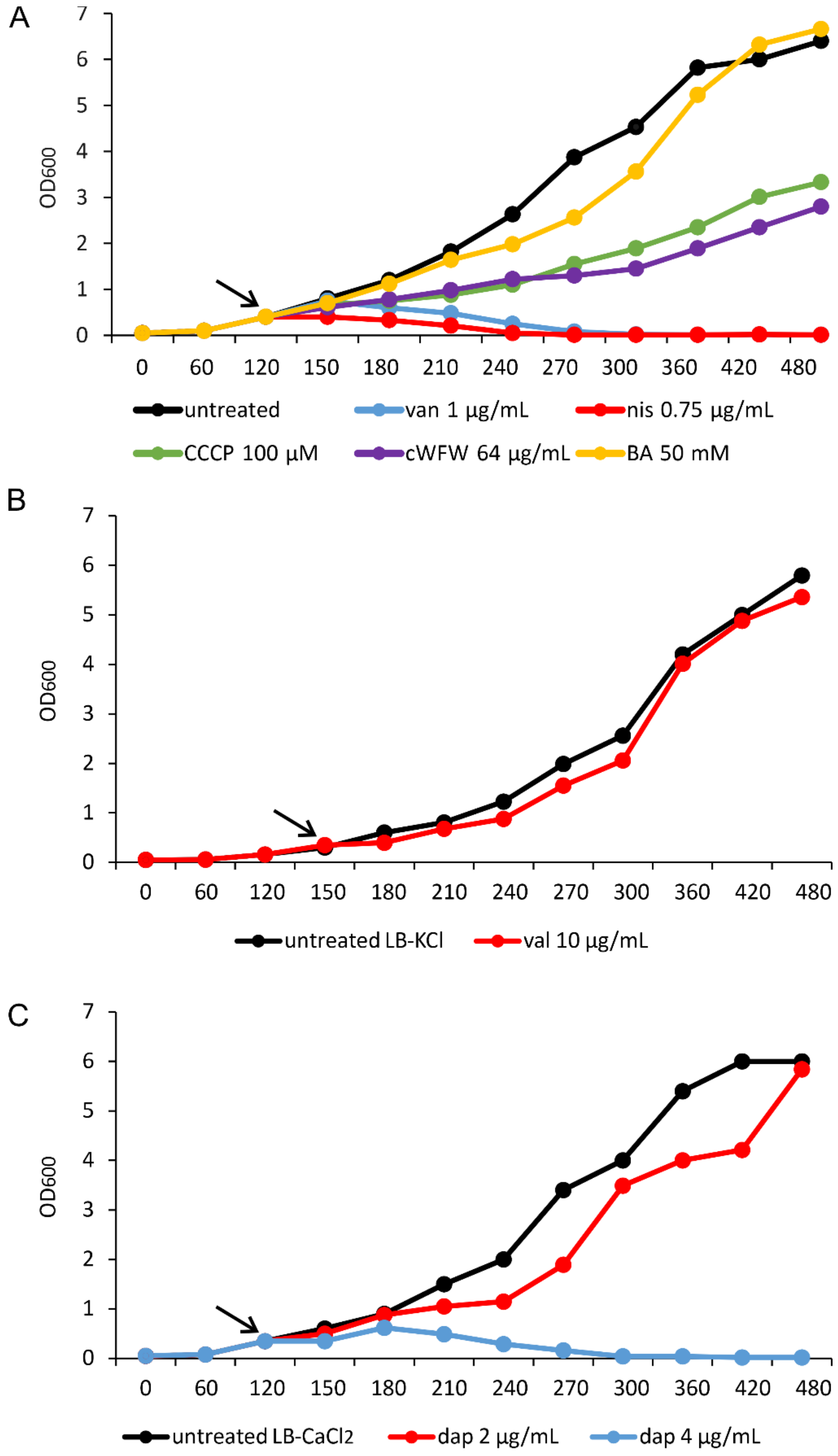

Figure S26: Growth of *B. subtilis* 168CA treated with antibiotics. Cultures were grown until early log phase prior to addition of antibiotics. (nis: nisin, van: vancomycin, CCCP: carbonyl cyanide m-chlorophenyl hydrazone, cWFW: cRRRWFW, BA: benzyl alcohol, dap: daptomycin, val: valinomycin). In contrast to other antibiotics **(A)**, daptomycin **(B)** and valinomycin **(C)** require supplementation with 1.25 mM $CaCl_2$ and 300 mM KCl, respectively, and thus have separate untreated controls. Arrows indicate timepoint of antibiotic addition.

References

1. Spizizen J. 1958. Transformation of Biochemically Deficient Srains of *Bacillus subtilis* by deoxyribonucleate. Proc Natl Acad Sci 44:1072–1078.
2. Aguilar PS, Hernandez-Arriaga AM, Cybulski LE, Erazo AC, de Mendoza D. 2001. Molecular basis of thermosensing: a two-component signal transduction thermometer in *Bacillus subtilis*. EMBO J 20:1681–91.
3. Koo B-M, Kritikos G, Farelli JD, Todor H, Tong K, Kimsey H, Wapinski I, Galardini M, Cabal A, Peters JM, Hachmann A-B, Rudner DZ, Allen KN, Typas A, Gross CA. 2017. Construction and Analysis of Two Genome-Scale Deletion Libraries for *Bacillus subtilis*. Cell Syst 4:291-305.e7.
4. Hachmann AB, Angert ER, Helmann JD. 2009. Genetic analysis of factors affecting susceptibility of *Bacillus subtilis* to daptomycin. Antimicrob Agents Chemother 53:1598–1609.
5. Müller A, Wenzel M, Strahl H, Grein F, Saaki TN V., Kohl B, Siersma T, Bandow JE, Sahl H-G, Schneider T, Hamoen LW. 2016. Daptomycin inhibits cell envelope synthesis by interfering with fluid membrane microdomains. Proc Natl Acad Sci 113.
